# An image partition security-sharing mechanism based on blockchain and chaotic encryption

**DOI:** 10.1371/journal.pone.0307686

**Published:** 2024-07-29

**Authors:** Na Wang, Xiaochang Wang, Aodi Liu, Wenjuan Wang, Yan Ding, Xiangyu Wu, Xuehui Du

**Affiliations:** PLA Information Engineering University, Zhengzhou, Henan, China; Najran University College of Computer Science and Information Systems, SAUDI ARABIA

## Abstract

To ensure optimal use of images while preserving privacy, it is necessary to partition the shared image into public and private areas, with public areas being openly accessible and private areas being shared in a controlled and privacy-preserving manner. Current works only facilitate image-level sharing and use common cryptographic algorithms. To ensure efficient, controlled, and privacy-preserving image sharing at the area level, this paper proposes an image partition security-sharing mechanism based on blockchain and chaotic encryption, which mainly includes a fine-grained access control method based on Attribute-Based Access Control (ABAC) and an image-specific chaotic encryption scheme. The proposed fine-grained access control method employs smart contracts based on the ABAC model to achieve automatic access control for private areas. It employs a Cuckoo filter-based transaction retrieval technique to enhance the efficiency of smart contracts in retrieving security attributes and policies on the blockchain. The proposed chaotic encryption scheme generates keys based on the private areas’ security attributes, largely reducing the number of keys required. It also provides efficient encryption with vector operation acceleration. The security analysis and performance evaluation were conducted comprehensively. The results show that the proposed mechanism has lower time overhead than current works as the number of images increases.

## 1. Introduction

The world has entered the era of big data due to the rapid development and wide application of information technology [1–5]. The sharing and application of image data is becoming increasingly widespread, providing greater convenience to people’s lives. For instance, self-driving systems trained on real-time car images of road conditions have the potential to make driving safer and easier for drivers. Currently, the mainstream solution for data sharing [6–14] is based on blockchain technology due to its characteristics, such as open-sharing, distributed, non-tampering, traceable, and programmable.

**Fig 1:**
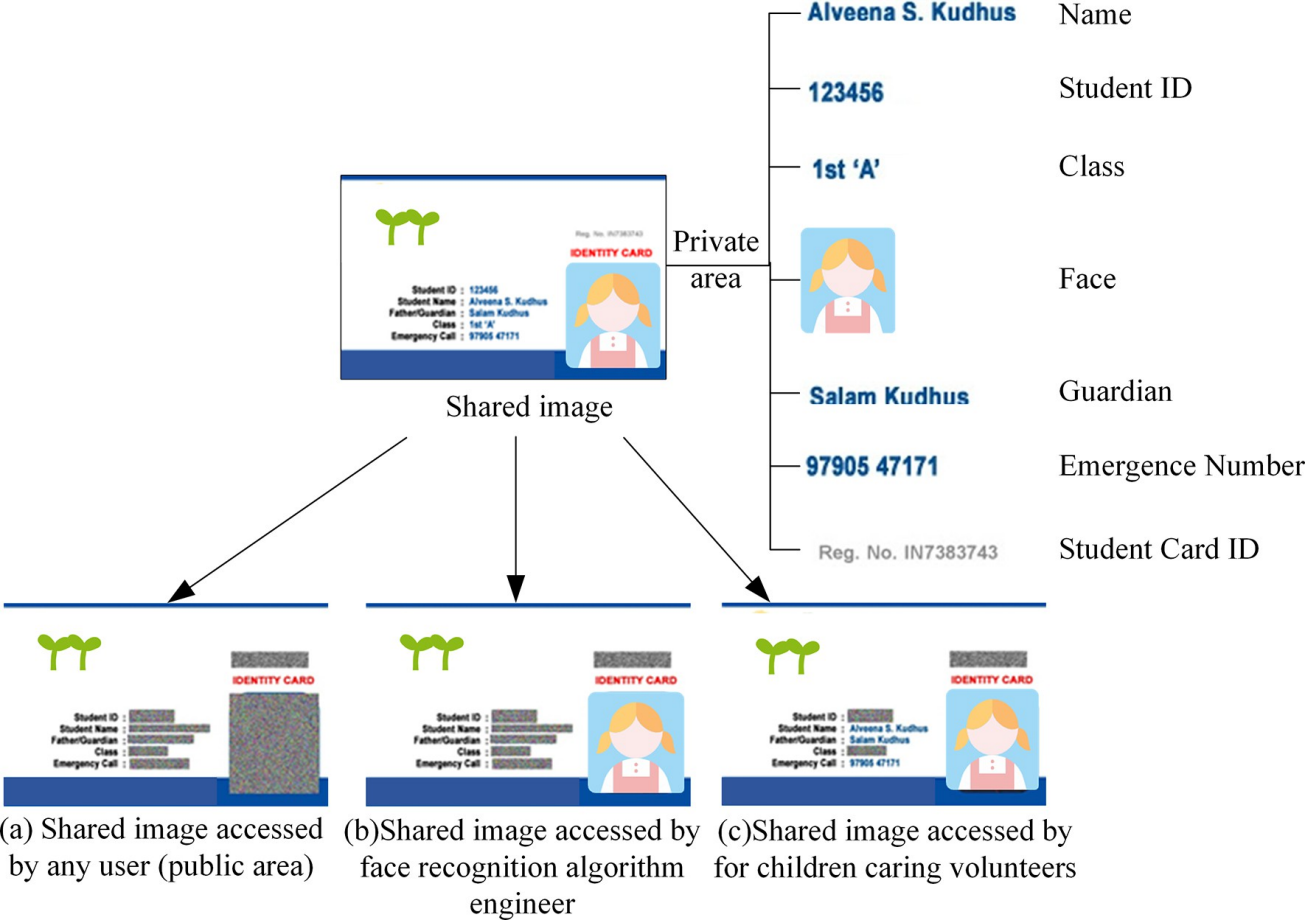
An example of partition security sharing of images.

However, blockchain-based image-sharing mechanisms currently only allow for sharing at the ‘image-level’ and not at a more granular ‘area-level’. This limitation may result in the exposure of personal private information in the image, posing significant risks [15, 16]. To ensure optimal use of images while preserving privacy, it is essential to partition the image into public and private areas. Public areas are available to all users, while private areas are shared in a controlled and privacy-preserving manner through access control and encryption. Only users who meet the security policy requirements can decrypt and access these areas. For example, Fig 1 displays seven private areas: ‘Name’, ‘Student ID’, ‘Class’, ‘Face’, ‘Guardian’, ‘Emergency Number’, and ‘Student Card ID’. The public area of the image (as shown in Fig 1A) is accessible to any user. The ‘Face’ private area can only be accessed by the face recognition algorithm engineer with the user’s permission, as shown in Fig 1B. For children caring volunteers, the accessible private areas are ‘Face’, ‘Name’, ‘Emergency Number’, and ‘Guardian’, as shown in Fig 1C.

In addition, current mechanisms typically use common cryptographic algorithms, such as AES, to encrypt shared images. However, image data is different from text data in that it is spatially ordered, has high redundancy and strong correlation [17, 18]. As a result, common cryptographic algorithms for text data are not suitable for encrypting image data [19]. Therefore, it is necessary to adopt a special image encryption algorithm to protect private areas of shared images.

This paper proposes an image partition security-sharing mechanism based on blockchain and chaotic encryption (IPSS_BCE) to achieve efficient, controlled, and privacy-preserving image sharing at the area level.

Specifically, the main contributions of this paper are as follows:

1. The fine-grained access control method based on ABAC is proposed to achieve efficient and automatic access control of shared private areas.
  The ABAC model is employed in the proposed method to make access decisions based on the security attributes of users and private areas, ensuring fine-grained access control at the area level. Meanwhile, smart contracts for Policy Information Point (PIP), Policy Administration Point (PAP), and Policy Decision Point (PDP) are designed to provide automatic access control. Furthermore, an on-chain transaction retrieval method based on the Cuckoo filter is proposed to enhance the retrieval efficiency of the smart contracts for access control policies and user security attributes on the blockchain.
2. The security attribute-related chaotic encryption scheme is proposed to protect private areas.
  This paper exploits the ‘chaotic image encryption’ scheme to protect private areas, in contrast to current mechanisms that use common cryptographic algorithms. However, it is noted that the key generation method of the scheme is ‘one image, one key’, indicating that different images are encrypted with different keys in the chaotic encryption scheme. In the scenario of image partition security sharing, there are numerous shared images, each with multiple private areas. If the ‘one image, one key’ key generation method is adopted, each private area is encrypted with a different key. This may result in a large-scale key management issue. However, when it comes to sharing private areas with fine-grained access control, it is unnecessary to encrypt them with different keys if they have the same security attribute. This is because, as long as a user meets the access control policy, he can legitimately access all private areas with the same security attribute that are constrained by the policy.
  The security attribute-related chaotic encryption scheme proposed in the paper generates keys based on the image’s security attributes to reduce the number of keys effectively. Also, the scheme adopts the two-dimensional chaotic system 2D-HSM [20] and the five-dimensional multi-ring multi-wing hyperchaotic system [21] to randomly generate the key matrix and the initial parameters based on the key. Besides, the proposed scheme employs the diffusion encryption method of plain image correlation and vector operation acceleration. It generates the plain-related initial diffusion positions to ensure the plaintext sensitivity of the scheme, and it uses vector operations to accelerate the full diffusion process of the key matrix on the plain image matrix, thereby improving the encryption and decryption speed of the scheme.
3. The on-chain and off-chain collaborative sharing architecture and the whole image partition security-sharing process are presented in the paper. The proposed mechanism adopts the same on-chain and off-chain collaborative approach as current research works to address the issue of small storage capacity on the blockchain, but it defines a special storage format on the off-chain system and shared metadata on the blockchain.
4. The paper presents a comprehensive security analysis and performance evaluation. The study demonstrates that the proposed encryption scheme has a positive impact on image encryption and has low time complexity. Furthermore, it significantly reduces the number of keys. Additionally, the proposed mechanism meets the security requirements for image partition sharing. The study also found that the proposed transaction retrieval method based on the Cuckoo filter has higher retrieval efficiency than the method based on the Bloom filter. Finally, the proposed mechanism has lower time overhead than current works as the number of shared images increases.

The rest of the paper is organized as follows. Section 2 discusses the related work. Section 3 describes the on-chain and off-chain collaborative sharing architecture of the proposed IPSS_BCE mechanism. Sections 4 and 5 introduce the proposed efficient fine-grained access control method based on ABAC and the security attribute-related chaotic encryption scheme, respectively. Section 6 provides the whole process of image partition security-sharing. Section 7 performs a detailed security analysis of the IPSS_BCE mechanism, and Section 8 presents the performance evaluation details. Finally, Section 9 concludes the paper.

## 2. Related work

This section presents a detailed analysis of the current research status of blockchain-related image sharing technology and image encryption algorithms based on chaotic systems, and also highlights the limitations of current research.

### 2.1 Blockchain-based image-sharing technology

Due to the storage structure and the high-redundancy storage mode of the blockchain, shared images cannot be directly stored on the blockchain. Therefore, a blockchain-based image-sharing mechanism stores the shared image off the chain and only stores the metadata of the shared image on the chain. Meanwhile, it adopts access control methods to control image sharing, exploits smart contracts to automate control of users’ access to the shared image, and uses encryption algorithms to protect the privacy of shared images. This section mainly analyzes current research on blockchain-based image-sharing technology from the aspects of off-chain storage methods, access control methods, encryption methods, etc.

In the blockchain-based medical image data-sharing framework [12], the medical images of patients are stored on the local database of the hospital, and a URL access link for each medical image is stored on the blockchain. An Access Control List (ACL) is established for each patient, and it is stored on the blockchain to prevent malicious tampering. When a doctor requests a patient’s medical image, the connection can be successfully established to access the medical image if the doctor is in the patient’s ACL. In the Fabric-iot scheme [13], shared data is stored locally on IoT devices, while the URL of the shared data, user access control policies, and other relevant information are stored on the blockchain. Access control is based on the ABAC model, but only considering the security attributes of users. Furthermore, the Fabric-iot scheme designed three types of smart contracts, namely Device Contract (DC), Policy Contract (PC), and Access Contract (AC), to automate control over user access to resources. However, it is noteworthy that the methods referenced in [22, 23] result in high storage overhead, as all shared image is locally stored on hospital databases or IoT devices.

In the PrivySharing scheme [24], the blockchain network is divided into multiple channels, each composed of a limited number of authorized organizations to protect data privacy. Each channel is responsible for processing specific types of data, such as health, smart car, or smart meter data. Meanwhile, certain critical data is further encrypted using the AES-256 algorithm. Access control to users’ data within the channels is enforced by embedding ACL in smart contracts. However, it does not address the storage of unstructured data (such as medical images or surveillance images), and employ common cryptographic algorithm to encrypt images.

A method for medical image sharing based on blind digital certificates and Merkle trees has been proposed in reference [25]. This method utilizes a cloud server to store the cipher image, employs a symmetric encryption algorithm for identity authentication and image privacy protection, and leverages blind digital certificates for efficient symmetric key distribution. Meanwhile, authentication between patients and medical analysis devices is achieved with the Merkle authentication model and smart contracts. However, access control for shared images has not been considerd in this method. The reference [26] proposed a mechanism called MedSBA for secure medical data sharing based on blockchain and cloud storage, which also stores cipher images on cloud servers. The AES algorithm is utilized to encrypt the medical image, while the Attribute-Based Encryption (ABE) algorithm is used to encrypt the AES key. This ensures that only users with appropriate attributes can decrypt and obtain the key, providing access control and privacy of shared images. The mechanism adopts a permissioned blockchain and a non-permissioned blockchain, where the cipher image’s storage path in the cloud is on the permissioned blockchain, and the description and access conditions for shared image are on the non-permissioned blockchain. The secure sharing technique called Sash for IoT data [27] provides two sharing control methods: ACL and prefix encryption. Prefix encryption is a variant of hierarchical identity-based encryption. In the prefix encryption method, the cipher image is stored on a cloud server and the hierarchical relationship between visitors’ names is leveraged to recover the image encryption key, thereby enabling the controlled sharing of images. Also, this technique adopts smart contracts to automate access control of shared images.

The reference [28] presented a blockchain-based data-sharing scheme for vehicle social networks. This scheme employs Ciphertext-Policy Attribute-Based Encryption (CP-ABE) to ensure secure one-to-many data sharing. Specifically, the ciphertext is stored on a cloud server, while the access control policy, the hash of the shared data, and the signature of the cloud server are stored on the blockchain. Meanwhile, it provides support for hiding sensitive information in access policy and facilitates data revocation when necessary. However, the methods referenced in [25–28] employ common cryptographic algorithms, such as AES and CP-ABE, for image encryption. The method described in [28] even utilizes the CP-ABE algorithm to directly encrypt the image, incurring a significant performance overhead. Meanwhile, these methods incorporate of a third-party cloud server, which raises concerns about the system’s distributed nature. Besides, the cloud server, as a semi-honest third party [29], has inherent vulnerabilities, such as privacy leakage and single points of failure. These drawbacks should be carefully considered when evaluating the effectiveness and security of the proposed scheme.

The InterPlanetary File System (IPFS) is a content-addressing-based distributed file system that assigns the hash value of a file as its storage address, thereby ensuring tamper-proof and deduplication. In comparison to cloud servers, the IPFS system has obvious advantages in terms of upload and download time overhead, especially when dealing with images [30]. To exploit these benefits, the reference [20] proposed a patient-centered medical image management mechanism that combines blockchain and IPFS technologies. The mechanism uses AES to encrypt images, and the cipher image is stored on the IPFS system. The IPFS address of cipher image, patient address, timestamp, and other relevant information are stored on the Ethereum platform. Only clinicians, medical institutions, insurance companies, etc. approved by patients can access the images, and smart contracts are employed to automatethe whole access control process. In the neuroimaging sharing scheme [31], the patients’ neuroimages are stored on the IPFS system. The IPFS address of the neuroimages, along with the patient ID, disease, and medication details, are recorded on the blockchain. To access the neuroimaging data, an automatic retrieval process is initiated from the IPFS system using a smart contract based on the IPFS address on the blockchain. However, this scheme does not achieve privacy protection or access control for the users’ neuroimages. The reference [32] introduced a secure medical image-sharing system based on blockchain and the zero-trust principle. By leveraging the zero-trust principle, the system provides strong login authentication for both image senders and receivers. Access control to shared images is based on the Role-Based Access Control (RBAC) model. The shared images are encrypted with symmetric cryptographic algorithm, and their cipher images are stored in the IPFS system. Then, the corresponding storage addresses of these cipher images are recorded on the blockchain. However, storing the encryption keys in plaintext on the blockchain poses a security threat of key leakage. Additionally, the reference [33] proposed an IoT data-sharing scheme by combining CP-ABE and blockchain technology. The image is encrypted using the AES algorithm, and the resulting cipher image is stored in the IPFS system. Meanwhile, CP-ABE is utilized to encrypt the encryption keys to ensure that only users who satisfy the access control policy can obtain these keys. The data hash value, ciphertext storage address, and access control policy are stored on the blockchain. Besides, smart contracts are adopted to enforce access control. However, the methods referenced in [30, 32, 33] also employ common cryptographic algorithms for image encryption. The method in reference [33] introduces a centralised key distribution authority for generating key pairs for the CP-ABE algorithm, which compromises the distributed nature of the system.

The reference [34] introduced a blockchain-based framework for secure image sharing, which employs reversible data hiding and encryption techniques to safeguard images. Access control to shared data is based on the RBAC model, where an image can be shared only when more than 50% of administrators grant permission. However, this framework primarily focuses on discussing access control and reversible data-hiding mechanisms. The reference [35] presented a blockchain-based secret image sharing (SIS) scheme. SIS shares a secret image by generating *n* shadow images, and any subset of *k* shadow images can be used to reconstruct the secret image. This scheme encrypts the shadow images using fully homomorphic encryption algorithms and stores them in an off-chain IPFS system. Meanwhile, the storage addresses are stored on the blockchain. Besides, a smart contract for identity authentication is deployed to achieve the (*k*, *n*) threshold for secret image restoring. Outsourcing computation is utilized to reduce the computational burden on smart contracts and users for secret image restoring. However, it should be noted that this scheme requires the permission of at least *k* participants to restore the secret image and has limited applicability in certain scenarios.

In summary, current blockchain-based image sharing mechanisms only achieve controlled sharing at the image level based on the user’s identity, roles, or security attributes. This paper addresses this limitation by proposing the area-level fine-grained access control method based on both the user’s and private area’s security attributes. Meanwhile, these mechanisms employ common cryptographic algorithms for image encryption, and some don’t even protect the image encryption key. To tackle the issue, this paper proposes an image-specific chaotic encryption scheme to encrypt private areas and provides protection to image encryption keys. Table 1 shows a detailed comparison of related works.

**Table 1:** Summary of related work.

Ref.	Off-chain storage	Access Control	Control basis	Control granularity	Image encryption	Key protection	Smart contract	Fully distributed
**[22]**	Local database	ACL	User’s identity	Image level	_	_	_	Yes
**[23]**	IoT device	ABAC	User’s attribute	Image level	_	_	Yes	Yes
**[24]**	_	ACL	User’s identity	Image level	AES	_	Yes	Yes
**[25]**	Cloud server	_	_	_	Symmetric encryption	Blind digital certificate	Yes	No
**[26]**	Cloud server	ABE	User’s attribute	Image level	AES	ABE	Yes	No
**[27]**	Cloud server	ACL/prefix encryption	User’s identity	Image level	Symmetric encryption	Prefix encryption	Yes	No
**[28]**	Cloud server	ABE	User’s attribute	Image level	CP-ABE	_	Yes	No
**[30]**	IPFS	ACL	User’s identity	Image level	AES	Asymmetric encryption	Yes	Yes
**[31]**	IPFS	_	_	Image level	_	_	Yes	Yes
**[32]**	IPFS	RBAC	User’s Role	Image level	Symmetric encryption	_	Yes	Yes
**[33]**	IPFS	CP-ABE	User’s attribute	Image level	AES	CP-ABE	Yes	No
**[34]**	_	RBAC	User’s Role	Image level	Reversible data hiding and encryption	_	_	_
**[35]**	IPFS	_	_	Image level	(*k*,*n*) Secret image sharing	_	Yes	Yes

### 2.2 The image encryption algorithms based on chaotic system

**Fig 2:**
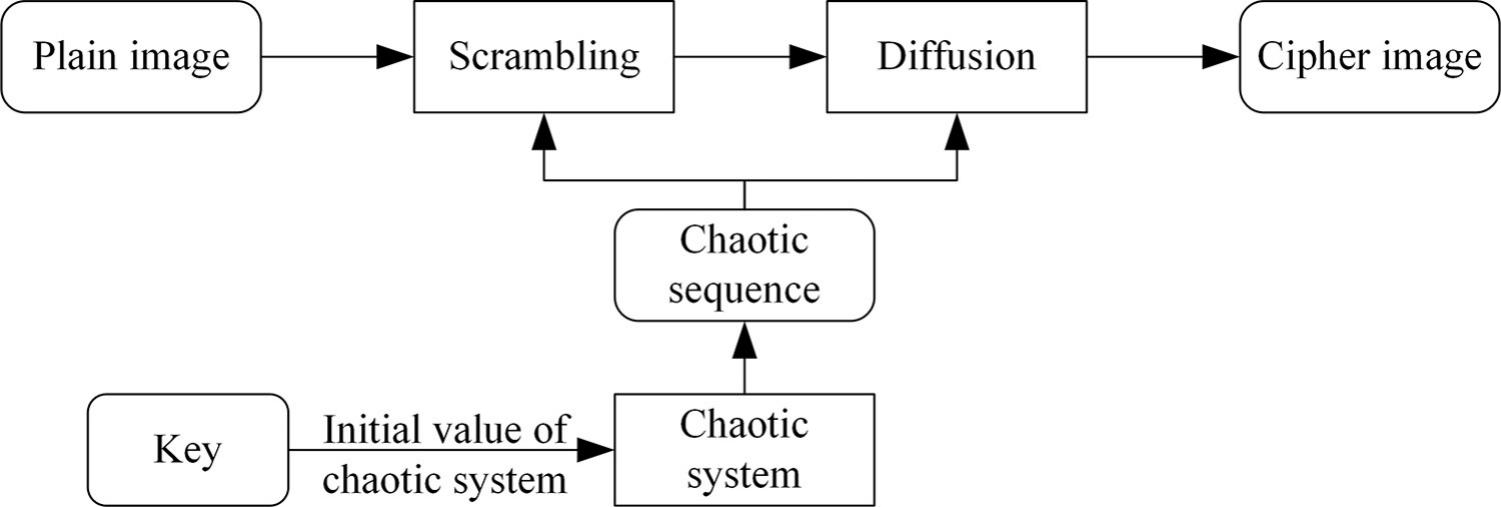
The flow chart of the chaotic encryption algorithm.

The current image encryption algorithms mainly include the encryption algorithms based on Discrete Cosine Transform (DCT) [36, 37] and chaotic encryption algorithms [38–44]. The DCT-based encryption algorithm encrypts the image by breaking the correlation between image pixels, which is achieved by setting the privacy threshold *T* and differently processing the lower and higher DCT coefficients than *T*. However, this encryption algorithm is only applicable for JPEG images and has weak security [45]. The chaotic encryption algorithm utilizes a chaotic system to generage a random chaotic sequence, taking advantage of its sensitive initial values, non-periodicity, and good randomness. It then encrypts the image by scrambling and diffusing the image matrix with the random chaotic sequence. As a result, the image’s statistical characteristics have been changed, resulting in a more uniform pixel distribution. The flow chart of the chaotic encryption algorithm is shown in Fig 2.

The reference [38] proposed a chaotic image encryption algorithm based on Latin square and random shift. The algorithm divides the image matrix into four parts, and the sum of pixels in each part is used as the initial value of the four-dimensional Lorenz hyperchaotic system to generate chaotic sequences. Meanwhile, each pixel in each row of the image matrix is rotated to the right, and the cyclic shift step is controlled by the chaotic sequence to realize pixel position scrambling. The Latin square array generated according to the chaotic sequence is used to replace the pixels and scramble the bits of the image to generate the cipher image. The reference [40] proposed a plaintext-related chaotic image encryption scheme on compressive sensing to realize compression and encryption simultaneously. The scheme generates keys based on the SHA256 hash value of the image. It then convert the image to the coefficient matrix using discrete wavelet transform and conducts permutation processing on the coefficient matrix. This is controlled by the two-dimensional sine improved logistic iterative chaotic system to generate the measurement matrix. As a result, the plaintext-related compressive sensing is achieved. It performs the permutation and diffusion operation by the two-dimensional logistic-sine-coupling system to obtain the cipher image. The reference [43] proposes an image encryption algorithm based on the improved Arnold transform and chaotic pulse-coupled neural network. The algorithm generates chaotic sequences through multiple iterations of the chaotic pulse-coupled neural network with the predefined key. It then performs XOR operations on the chaotic sequence and plain image to generate the pre-encrypted image. Finally, it scrambles the pre-encrypted image by the improved Arnold transform to obtain the final cipher image.

It is founded that the current key generation methods used in the chaotic encryption algorithms mainly include static predefinition [43] and dynamic generation based on characteristic values of plain images, such as pixels sum [38], SHA256 value [40], etc. Compared with the predefined key generation method, the key generation method based on the characteristic value of the plain image is more sensitive to the plaintext. This is because the characteristic values are different for each plain image. The resistance to differential attacks increases with the strength of the plaintext sensitivity. However, both of the two key generation methods are “one image, one key”, indicating the chaotic encryption algorithm encrypts different images with different keys. In the context of this study, if a user’s security attributes meet the subject security attributes in the access control policy, he can legitimately access all private areas with the object security attributes in the policy. In this case, if the “one image, one key” key generation method is still used, each private area of each shared image will have its own key. If there are multiple private areas in a shared image and the number of shared images is large, a significant number of keys will be required. This will increase the complexity and workload of key distribution between image sharers and users. As a result, the current “one image, one key” key generation method is not suitable for the image partition security sharing scenario in this paper. To address this issue, this paper proposes a security attribute-related chaotic encryption scheme, which generates keys based on the security attributes of private areas to effectively reduce the number of keys.

## 3. On-chain and off-chain collaborative sharing architecture

**Fig 3:**
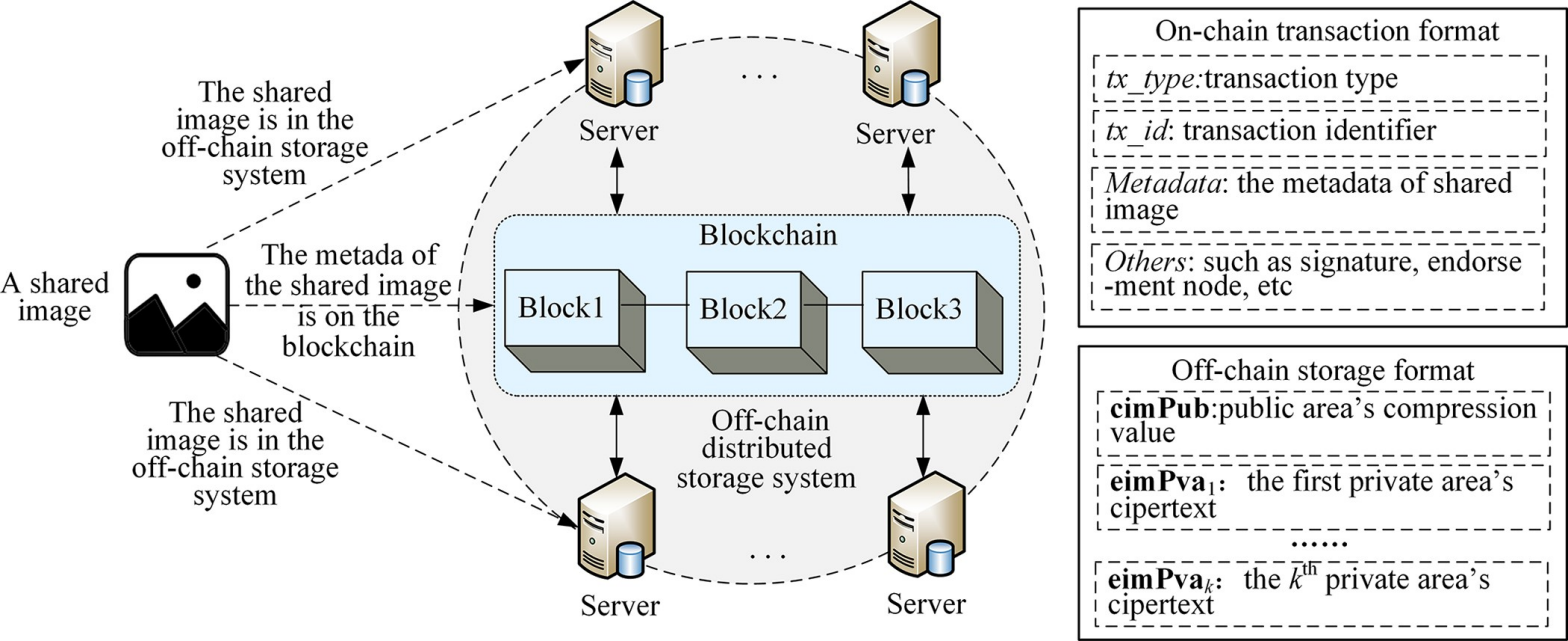
The on-chain and off-chain collaborative sharing architecture.

The on-chain and off-chain collaborative sharing architecture is designed in the proposed IPSS_ BCE mechanism to address the issue of limited storage capacity on the blockchain. Fig 3 illustrates that the shared image is stored in an off-chain distributed storage system, while the metadata of the shared image is recorded on the blockchain in the form of transactions. The off-chain distributed storage system comprises multiple servers, which can be established using distributed protocols such as IPFS. The off-chain storage format for shared images consists of the compression value of the public area and ciphertexts of the private areas of the image.

The metadata *Metadata* of the shared image includes the unique identifier of the image *ID*, the image fingerprint set *fdata*, the off-chain storage address *Faddr*, the address of the image sharer *Addr*, the security attribute set of the private areas *Attrs*, the pixel location set of the private areas *Boxes*, and the encryption parameter set of the private areas *EConfigs*:

Metadata={ID,fdata,Faddr,Addr,Attrs,Boxes,EConfigs} (1)

where $fdata=\{fPub,fPva_{1},\ldots ,fPva_{i},\ldots ,fPva_{k}\}$, *fPub* is the fingerprint of the public area, *fPva*_*i*_ is the fingerprint of the private area *i* (1≤*i*≤*k*, *k* is the number of private areas in the image). These fingerprints are generated by the image hash operation [46–48] on the image compression value, and they are stored on the blockchain to determine whether the shared image on the off-chain storage system has been tampered with. This ensures the consistency of the off-chain shared image and its on-chain metadata. In the Eq (1), *Attrs* ={*aPva*_1_,…,*aPva*_*k*_}, *Boxes* = {*Box*_1_,…,*Box*_*k*_}, *EConfigs* = {*EConfig*_1_,…,*EConfig*_*k*_}, where *aPva*_*i*_ is the security attribute of the private area *i*, *Box*_*i*_ is the pixel position of the private area *i*, and *EConfig*_*i*_ is the encryption parameters of the private area *i* (1≤*i*≤*k*).

After retrieving the shared metadata *Metadata* from the blockchain, users can obtain the image in off-chain storage format from the off-chain distributed storage system using the storage address *Faddr*. Then, the image public area can be accessed without any access control process, and users can verify whether the public area on the off-chain distributed storage system has been tampered with by checking its image fingerprint *fPub* in the *fdata*.

## 4. Efficient fine-grained access control method based on ABAC

The efficient fine-grained access control method based on ABAC is proposed in this paper to realize fine-grained and automatic access control of private areas in shared images. According to the security attributes of the subject, the security attributes of the object, the environmental conditions, and a set of access control policies based on the security attributes and conditions, the ABAC model allows or rejects the access requests of the subject [49]. The ABAC model is a dynamic, fine-grained, and scalable access control technology that is considered highly suitable for distributed scenarios [50–53]. This paper adopts the ABAC model to achieve fine-grained sharing of private areas and designs the PIP, PAP, and PDP contracts to automate access control to private areas. Besides, this paper proposes an on-chain transaction retrieval method based on the Cuckoo filter to enable the PIP contract and PAP contract to efficiently retrieve users’ security attributes and access control policies stored on the blockchain. This improves the efficiency of access control.

### 4.1 The ABAC-based access control smart contracts and access decision process

The proposed efficient fine-grained access control method based on ABAC relies on the following assumptions:

1. The subject’s security attribute space is *SSAttr* = {*SAttr*_1_,…, *SAttr*_*m*_}, where *SAttr*_*i*_ is the value set of the subject’s *i*^th^ attribute (1≤*i*≤*m*), *m* is the number of the subject’s security attribute;
2. The object’s security attribute space is *SOAttr* = {*OAttr*_1_,…, *OAttr*_*k*_}, where *OAttr*_*i*_ is the value set of the object’s *i*^th^ attribute (1≤*i*≤*k*), *k* is the number of the object’s security attribute;
3. The access control policy is well-defined, and there is a unique policy rule *CPRule* related to any subject’s attribute set *SAttrs* and any object’s attribute set *OAttrs*, where *SAttrs* = {*sattr*_1_,…, *sattr*_*m*_}, *sattr*_*i*_ is the value of the subject’s *i*^th^ attribute and *sattr*_*i*_∈*SAttr*_*i*_ (1≤*i*≤*m*), *OAttrs* = {*oattr*_1_,…, *oattr*_*k*_}, *oattr*_*i*_ is the attribute value of the object’s *i*^th^ attribute, and *oattr*_*i*_∈*OAttr*_*i*_(1≤*i*≤*k*), and the policy rule *CPRule* is represented as $sattr_{1}\wedge \ldots \wedge sattr_{m}\wedge oattr_{1}\wedge \ldots \wedge oattr_{k}\Rightarrow ALLOW/REJECT$.

The smart contracts of PIP, PAP, and PDP in the ABAC model are designed to provide automatic access control. The pseudocodes of the PIP, PAP, and PDP contracts are shown as follows.

**Algorithm 1 PIP_CONTRACT**: Retrieve the user’s security attributes based on their unique identifier from the attribute transaction set on the blockchain.

**Input**: *UID //* the user’s unique identifier

**Output**: *UAttrs //* the user’s security attributes

1. *Flag* = 0;

2. **For** *i* = blockchain.height() **to** *i* = 2 // traverse blocks from the back to the front

3. {

4.    *m* = this.block.blockdata.attrfilter;

5.    *p* = FNV_64(*UID*);

6.    *h*1 = hash(*UID*) mod *m*;

7.    *h*2 = (*h*1⊕hash(*p*)) mod *m*;

8.   **if** (*h*1 or *h*2 in *attrFilter*) //determine whether the *UID* exists in the attribute transaction

         set by the attribute Cuckoo filter.

9.    **then** {

10.               *UAttrs* = get_value(*UID*); // query key-value pairs to obtain user security

         attributes

11.               *Flag* = 1;

12.               break;

13.         }

14.    **else** *i*--;

15.}

16.**if** (*Flag* = 1)

17.**then return** *UAttrs*;

18.**else return** *NULL*.

**Algorithm 2 PAP_CONTRACT:** Retrieve the access control policy rule according to the security attribute of the private area from the policy transaction set on the blockchain.

**Input**: *OAttrs*_*pri*_ *//* the attribute set of the private area

**Output**: *Ptx*_*id* //the policy transaction identifier on the blockchain

              *PRule* // the access control policy rule related to *OAttrs*_*pri*_

1.*Flag* = 0;

2.**For** *i* = blockchain.height() **to** *i* = 2 // traverse blocks from the back to the front

3.{

4.    *m* = this.block.blockdata.policyfilter;

5.    $p=\mathrm{FNV}\_64(oattr_{1}^{pri}||\ldots ||oattr_{k}^{pri})$;

6.    $h1=hash(oattr_{1}^{pri}||\ldots ||oattr_{k}^{pri})\mathrm{mod}m$;

7.    *h*2 = (*h*1⊕hash(*p*)) mod *m*;

8.    **if** (*h*1 or *h*2 in *policyFilter*) //determine whether the *OAttrs*_*pri*_ exists in the policy

      transaction set by the policy Cuckoo filter.

9.    **then** {    

10.               *Ptx_id =* get_id; //obtain the policy transaction identifier

11.$PRule=\mathrm{get}\_\mathrm{value}(oattr_{1}^{pri}||\ldots ||oattr_{k}^{pri})$; // query key-value pairs to obtain the

         access control policy rule related to *OAttrs*_*pri*_

12.               *Flag* = 1;

13.               break;

14.             }

15.    **else** *i*--;

16. }

17. **if** (*Flag* = 1)

18. **then  return** *Ptx_id*, *PDetails*;

19. **else  return** *NULL*. 

**Algorithm 3 PDP_CONTRACT:** Make the access control decision.

**Input**: *UID*, *OAttrs*_*pri*_ *//* an access request including *UID* and *OAttrs*_*pri*_

**Output**: *RESULT* // the decision result

1.*UAttrs* = PIP_CONTRACT(*UID*) // request the user’s security attributes from the PIP contract

   according to the user’s unique identifier *UID*

2.*Ptx*_*id =* PAP_CONTRACT(*OAttrs*_*pri*_)

3.*PRule =* PAP_CONTRACT(*OAttrs*_*pri*_) // request the access control policy rule from the PAP

      contract according to the security attribute *OAttrs*_*pri*_

4.**For** *i* = 1 **to** *i≤*m

5. {**if** (*uattr*_*i*_|#*PRule*.*sattr*_*i*_) // *uattr*_*i*_ does not meet the corresponding subject’s security 

      attribute constraint in the access control policy rule *PRule*

6.  **then** { *RESULT*  = (*Ptx*_*id*, *UID*, *OAttrs*_*pri*_, *REJECT*);

7.             break; }

8.  **else** *i*++;

9.}

10.**if** (*i>m*)

11.**then** *RESULT* = (*Ptx*_*id*, *UID*, *OAttrs*_*pri*_, *ALLOW*);

12.**return** *RESULT*, *Rtx*_*id*.

**Fig 4:**
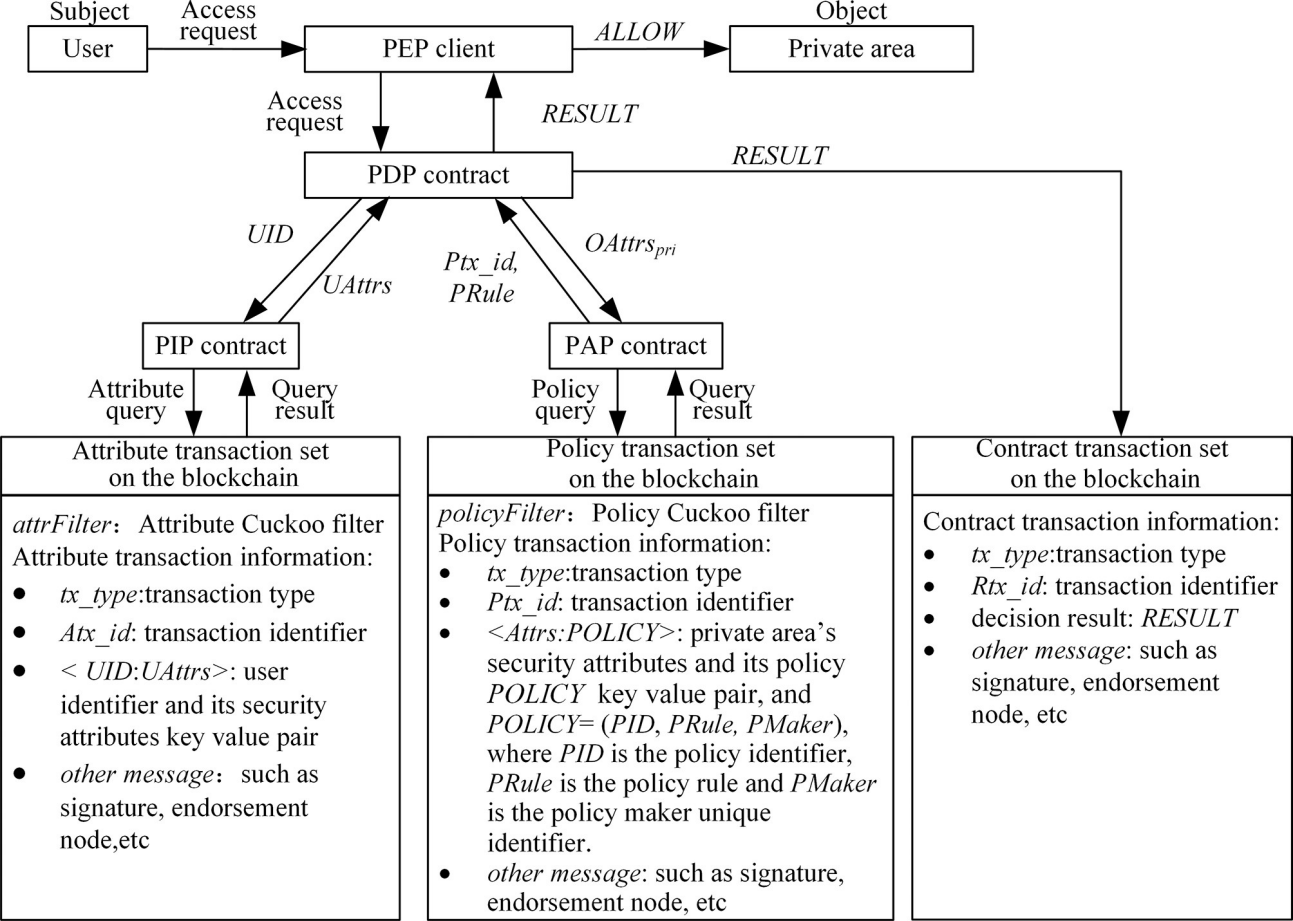
The access control process.

The access control process is shown in Fig 4. When a user sends an access request (*UID*, *OAttrs*_*pri*_), where *UID* is the user unique identifier, *OAttrs*_*pri*_ is the attribute set of the requested private area, and *$OAttrs_{pri}=\{oattr_{1}^{pri},\ldots ,oattr_{k}^{pri}\}$*, $oattr_{i}^{pri}$ is the value of the requested private area’s *i*^th^ attribute ($oattr_{i}^{pri}\in OAttr_{i}$, 1≤*i*≤*k*), the Policy Enforcement Point (PEP) client forwards the access request to the PDP contract. Then, the PDP contract requests the user’s attributes from the PIP contract based on *UID* and requests the policy rule related to *OAttrs*_*pri*_ from the PAP contract. After retrieving on the blockchain, the PIP contract returns the corresponding user attribute set *UAttrs* (*UAttrs* = {*uattr*_1_,…, *uattr*_*m*_}, *uattr*_*i*_∈*SAttr*_*i*_, 1≤*i*≤*m*), and the PAP contract returns the policy transaction ID *Ptx_id* and the policy rule *PRule* (*PRule* is *$sattr_{1}\wedge \ldots \wedge sattr_{m}\wedge oattr_{1}^{pri}\wedge \ldots \wedge oattr_{k}^{pri}\Rightarrow ALLOW/REJECT$*). The PDP contract makes a decision based on *UAttrs* and *PRule*. If the user’s attributes *UAttrs* meet the subject’s attribute constraints *sattr*_1_∧…∧*sattr*_*m*_ in *PRule*, access is allowed (*ALLOW*); otherwise, access is denied (*REJECT*). The decision *RESULT*, as shown in Eq (2), is recorded in the contract transaction set on the blockchain.


$$RESULT=\{Ptx\_id,UID,OAttrs_{pri},ALLOW/REJECT\}$$ (2)

### 4.2 On-chain transaction retrieval method based on the Cuckoo filter

The on-chain transaction retrieval method based on the Cuckoo filter is proposed to improve the retrieval efficiency of the PIP contract and PAP contract for user attributes and access control policies transactions on the blockchain. Compared with the Bloom filter [54], the Cuckoo filter has lower space overhead and higher retrieval efficiency.

The Cuckoo filter is an array of *M* buckets. Each bucket has *b* entries, and each entry holds the fingerprint of the key value *X*. The Cuckoo filter only stores the fingerprint of the key value, represented as *ϕ*_*X*_, which is generated by the FNV64 hash function. For the attribute transaction, *X* = *UID*, and for the policy transaction, $X=oattr_{1}^{pri}||\ldots ||oattr_{k}^{pri}$.

In the Cuckoo filter, each key value has two candidate buckets determined by hash functions *h*_1_(*X*) and *h*_2_(*X*), shown in Eq (3), where *hash*(•) is the Cuckoo hash function.


$$\begin{array}{l} h_{1}(X)=hash(X)\mathrm{mod}m\\ h_{2}(X)=(h_{1}(X)⊕hash(\phi_{X}))\mathrm{mod}m \end{array}$$ (3)

**Fig 5:**
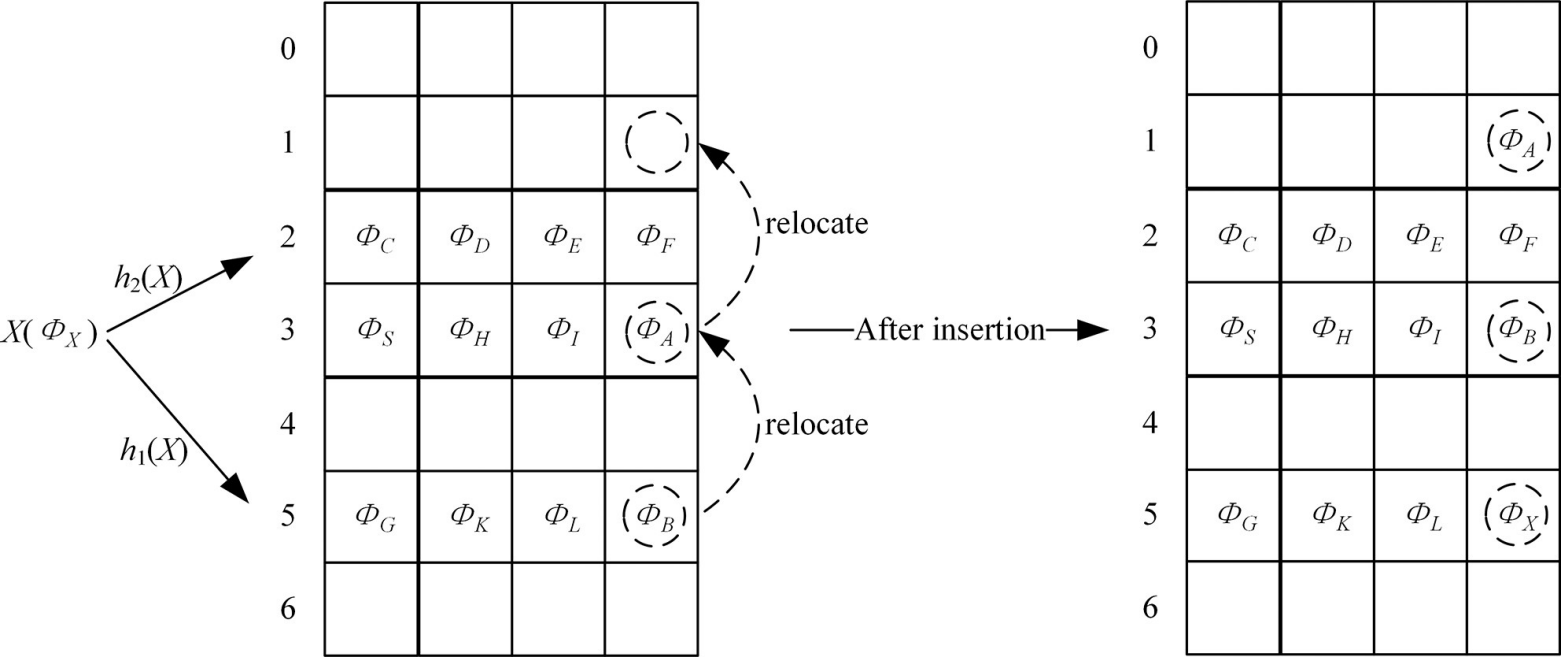
The method to construct the Cuckoo filter for the transactions on the blockchain.

The method to construct the Cuckoo filter for the transactions on the blockchain is described as follows (shown in Fig 5):

1. Calculate the bucket positions *h*_1_(*X*) and *h*_2_(*X*) of the key value *X*;
2. If the two buckets all have an empty entry, randomly select one bucket for inserting *ϕ*_*X*_;
3. If one of the two buckets has an empty entry, select the bucket with an empty entry for inserting *ϕ*_*X*_;
4. If the two buckets have no empty entry, randomly select an entry from the two buckets, swap *ϕ*_*X*_ and the fingerprint in the selected entry, and relocate the existing fingerprint until an empty entry is found or the number of relocations reaches the threshold. The relocation bucket position *e* is calculated as follows, and at the first time, *e* is *h*_1_(*X*) or *h*_2_(*X*).

$$e=e⊕hash(\phi_{X})\mathrm{mod}m$$ (4)

The method to retrieve the key value *X* in the Cuckoo filter is described as follows:

1. Calculate the bucket positions *h*_1_(*X*) and *h*_1_(*X*) of the key value *X*;
2. If any existing fingerprint in the two buckets matches *ϕ*_*X*_, the transaction set on the current block includes *X*. Take *X* as the key value, and retrieve its transaction data through the key-value pair querying in the transaction set.
3. If any existing fingerprint in the two buckets does not match *ϕ*_*X*_, the current transaction set does not include *X*. Go to the previous block to continue the retrieve process.
4. If all blocks on the blockchain do not contain the key value *X*, the retrieval process fails.

## 5. Security attribute-related chaotic encryption scheme

**Fig 6:**
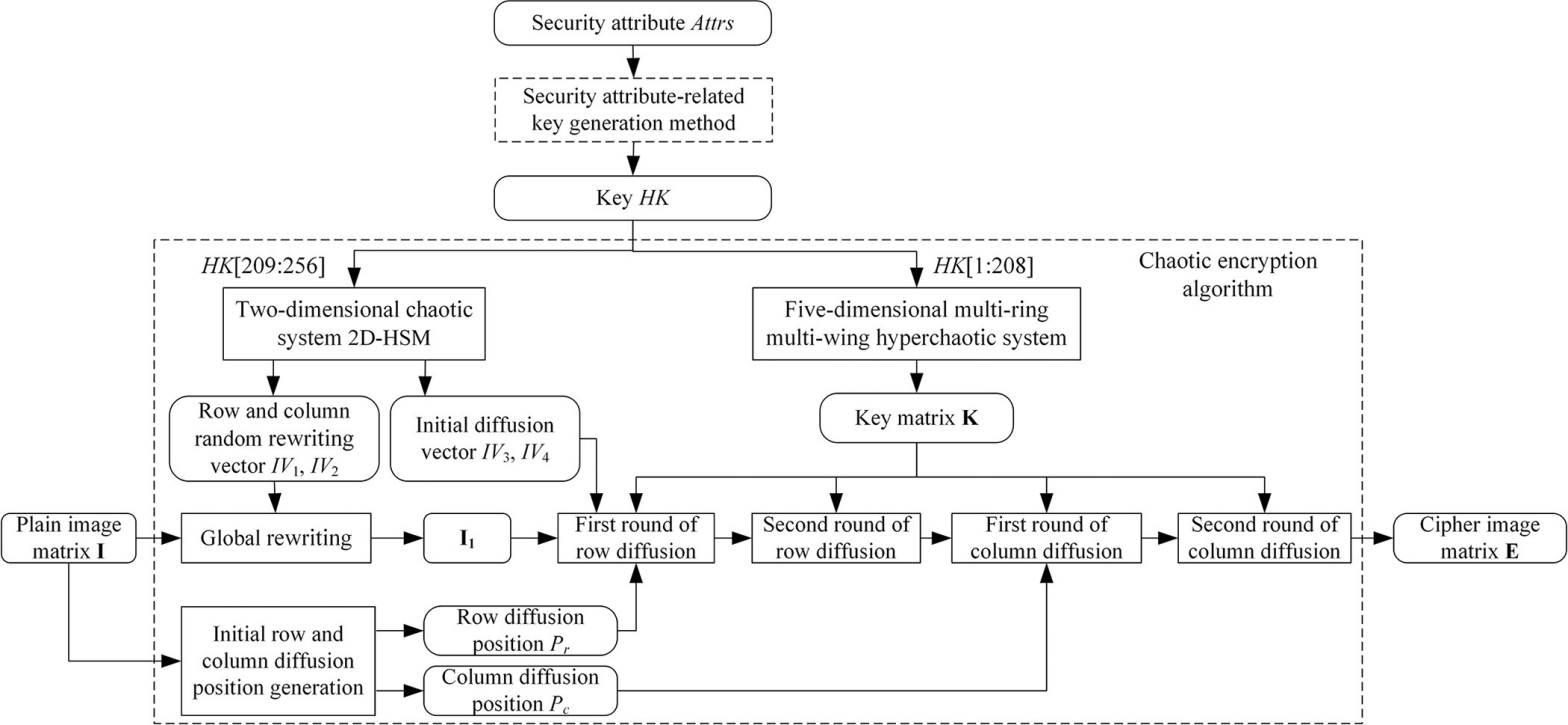
The block diagram of the proposed encryption scheme.

The proposed security attribute-related chaotic image encryption scheme includes the security attribute-related key generation method, the chaotic encryption algorithm with plain image correlation and vector operation acceleration, and the corresponding decryption algorithm (in S1 Appdendix in Supporting Information). The block diagram of the proposed encryption scheme is shown in Fig 6. The code of the proposed encryption scheme is provided in S1 File in Supporting Information.

### 5.1 Security attribute-related key generation method

The proposed security attribute-related key generation method generates the key based on the security attributes of the image. This method ensures that the same key is generated for private areas with the same security attributes, even if they are in the different shared images belonging to the same image sharer. As a result, the number of keys and key management burden can be reduced. Meanwhile, the proposed method introduces a random number in the key generation to avoid generating the same key for private areas with the same security attributes but belonging to different image sharers.

The key *HK* is calculated as follows:

$$HK=SHA256(attr_{1}||\ldots ||attr_{k})⊕R$$ (5)

where *SHA*256(•) represents the SHA256 hash function, *Attrs* = {*attr*_1_,…,*attr*_*k*_}, *attr*_*i*_∈*OAttr*_*i*_ (1≤*i*≤*k*), and *R* represents the 256-bit random number generated by the system. It is required that the random number *R* of the private areas with the same attributes in the same sharer is the same, and *R*≠*SHA*256(*attr*_1_‖…‖*attr*_*k*_).

### 5.2 Chaotic encryption algorithm with plain image correlation and vector operation acceleration

The proposed chaotic encryption algorithm first rewrites the entire plain image with random rewriting vectors to destroy the correlation between adjacent pixels in the plain image. Then, it performs the diffusion operation of plain image correlation and vector operation acceleration on the rewriting matrix with the key matrix to obtain the cipher image. The initial positions of the row and column diffusion is generated based on the statistical values of the image. This ensures that the proposed algorithm is plaintext-related, preventing a reduction in the plaintext sensitivity due to the key being independent of the plain image. Additionally, the diffusion process is accelerated by vector operations, which includes two rounds of two-vector parallel diffusion in the horizontal row direction and the vertical column direction respectively. As a result, the pixel information at any position in the plain image is efficiently diffused to all positions of the cipher image. Compared with the bit-level diffusion mode [55] and the pixel-level diffusion mode [56, 57], the vector diffusion mode can significantly accelerate the image encryption process. Compared with the common vector-level diffusion mode that performs only one vector diffusion at a time [58, 59], the vector diffusion mode in the paper has better randomness and unpredictability by performing two vector diffusions in parallel with two different random diffusion vectors.

In the proposed chaotic encryption algorithm, the two-dimensional chaotic system 2D-HSM [20] is adopted to generate random rewriting vectors and initial diffusion vectors, and the five-dimensional multi-ring multi-wing hyperchaotic system [21] is employed to generate the key matrix. The two chaotic systems have good chaotic performance, complex trajectories, well randomness, and enough random sequence space to meet the requirements of the algorithm in the paper.

The initial values of the two chaotic systems are calculated by the key *HK*. Due to the initial value sensitiveness and good randomness of chaotic system, even minor changes in the key will result in completely different rewriting vectors, initial diffusion vectors, and the key matrix. Therefore, the encryption algorithm in the paper is highly sensitive to the key.

**Fig 7:**
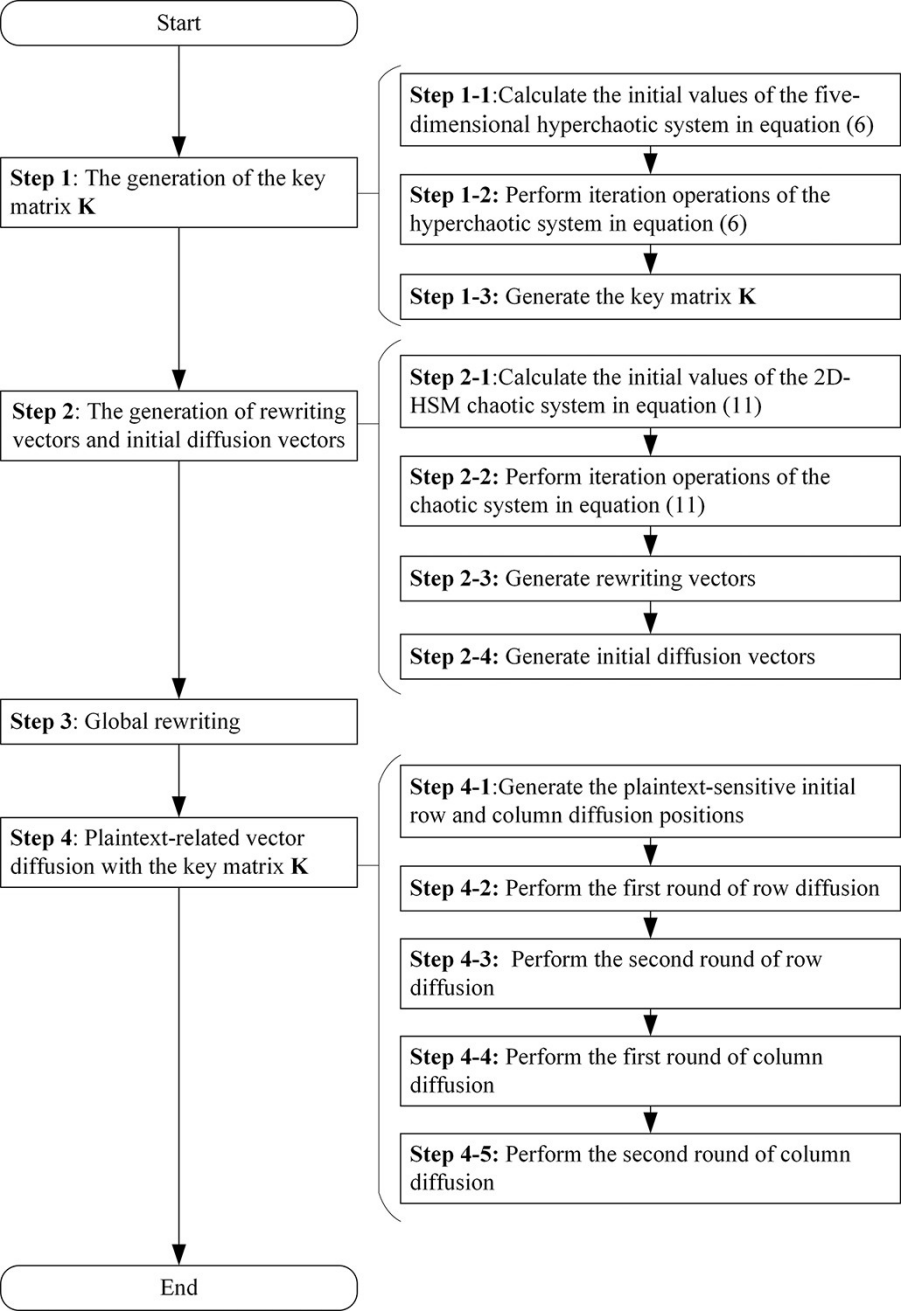
The flowchart of the proposed chaotic encryption algorithm.

As shown in Fig 7, the proposed algorithm encrypts the *M*×*N* image matrix **I** in the following manner:

### Step 1: The generation of the key matrix K

The key matrix **K** is generated by the five-dimensional multi-ring multi-wing hyperchaotic system [21] that uses the first 208-bit value (*HK*[1:208]) of the key *HK* as the initial value. The mathematical model of the five-dimensional multi-ring multi-wing hyperchaotic system is shown in Eq (6), where *step* is the iteration step, and *a*, *b*, *c*, *d*, *e*, *f*, and *g* are the parameters of the chaotic system.


$$\begin{array}{l} x_{i+1}-x_{i}=(ay_{i})\times step\\ y_{i+1}-y_{i}=(-bx_{i}+cy_{i}z_{i})\times step\\ z_{i+1}-z_{i}=(g-d{y_{i}}^{2})\times step\\ w_{i+1}-w_{i}=(e{y_{i}}^{3})\times step\\ v_{i+1}-v_{i}=(fz_{i})\times step \end{array}$$ (6)

The detailed steps are as follows:

**Step 1–1**: Calculate the initial values *x*_1_, *y*_1_, *z*_1_, *w*_1_, and *v*_1_ of the hyperchaotic system by the *HK* [1:200], as follows:

$$\begin{array}{l} x_{1}=T(HK[1:40])\times 2^{-40}\\ y_{1}=T(HK[41:80])\times 2^{-40}\\ z_{1}=T(HK[81:120])\times 2^{-40}\\ w_{1}=T(HK[121:160])\times 2^{-40}\\ v_{1}=T(HK[161:200])\times 2^{-40} \end{array}$$ (7)

where *T*(•) represents binary to decimal calculation. It is necessary to normalize the obtained decimal number because the initial value of the hyperchaotic system is between [0,1].

**Step 1–2**: Perform pre-iteration *n*_1_ times (*n*_1_ = *T*(*HK*[201:208])) to eliminate the potential safety hazard caused by the transient effect, and then continue iteration *n*_2_ times (*n*_2_ = (*M*×*N*)/5) to obtain the chaotic sequence matrix **C**, as follows.


$$C=\left[\begin{array}{cccccc} x_{1} & \ldots & x_{n_{1}} & x_{n_{1}+1} & \ldots & x_{n_{1}+n_{2}}\\ y_{1} & \ldots & y_{n_{1}} & y_{n_{1}+1} & \ldots & y_{n_{1}+n_{2}}\\ z_{1} & \ldots & z_{n_{1}} & z_{n_{1}+1} & \ldots & z_{n_{1}+n_{2}}\\ w_{1} & \ldots & w_{n_{1}} & w_{n_{1}+1} & \ldots & w_{n_{1}+n_{2}}\\ v_{1} & \ldots & v_{n_{1}} & v_{n_{1}+1} & \ldots & v_{n_{1}+n_{2}} \end{array}\right]$$ (8)

**Step 1–3**: Generate the key matrix **K**. Since the element values in the chaotic sequence matrix **C** are in the real domain and cannot be directly used for image encryption, it is necessary to quantify the element values in **C** to [0, 255], as shown in Eq (9), where ⌊ ⌋ represents the round-down operation.


$$C_{1}=\left\lfloor (|C|-\left\lfloor \left|C\right|\right\rfloor )\times 10^{15}\right\rfloor \mathrm{mod}\;256$$ (9)

Reconstruct the submatrix **C**_2_ shown in Eq (10), and substitute it into the *M*×*N* key matrix **K**.


$$C_{2}=\left[\begin{array}{ccc} x_{n_{1}+1} & \ldots & x_{n_{1}+n_{2}}\\ y_{n_{1}+1} & \ldots & y_{n_{1}+n_{2}}\\ z_{n_{1}+1} & \ldots & z_{n_{1}+n_{2}}\\ w_{n_{1}+1} & \ldots & w_{n_{1}+n_{2}}\\ v_{n_{1}+1} & \ldots & v_{n_{1}+n_{2}} \end{array}\right]$$ (10)

### Step 2: The generation of rewriting vectors and initial diffusion vectors

The 2D-HSM chaotic system is used to iterate the chaotic sequence with the initial value *HK*[209:256] to generate the random rewriting vectors (*Ⅳ*_1_, *Ⅳ*_2_) and the initial diffusion vectors (*Ⅳ*_3_, *Ⅳ*_4_).

The mathematical model of the 2D-HSM chaotic system is described as follows, where *α* and *β* are the parameters of the chaotic system.


$$\begin{array}{l} r_{i+1}=(1-\alpha \times \sin^{2}(r_{i})+s_{i})\mathrm{mod}\;1\\ s_{i+1}=(\beta r_{i})\mathrm{mod}\;1 \end{array}$$ (11)

The detailed steps are as follows:

**Step 2–1**: Generate the initial values *r*_1_ and *s*_1_ with *HK*[209:248] as follows:

$$\begin{array}{l} r_{1}=T(HK[209:228])\times 2^{-20}\\ s_{1}=T(HK[229:248])\times 2^{-20} \end{array}$$ (12)

**Step 2–2**: Perform pre-iteration *n*_3_ times (*n*_3_ = *T*(*HK*[249:256])), followed by iteration *n*_4_ times (*n*_4_ = max_*M*,*N*_+N, max_*M*,*N*_ = *Max*(*M*,*N*)) to generate vectors *Ⅳ* and *Ⅴ*, as follows.


$$\begin{array}{l} IV=[r_{1},\ldots ,r_{n_{3}},r_{n_{3}+1},\ldots ,r_{n_{3}+n_{4}}]\\ V=[s_{1},\ldots ,s_{n_{3}},s_{n_{3}+1},\ldots ,s_{n_{3}+n_{4}}] \end{array}$$ (13)

**Step 2–3**: Generate an *N*-dimensional row-rewriting vector *Ⅳ*_1_ and an *M*-dimensional column-rewriting vector *Ⅳ*_2_ with *Ⅳ* and *Ⅴ*, as follows.


$$\begin{array}{l} IV_{1}=IV[n_{3}+1:n_{3}+N]=[r_{n_{3}+1},\ldots ,r_{n_{3}+N}]\\ IV_{2}=V[n_{3}+1:n_{3}+M]=[s_{n_{3}+1},\ldots ,s_{n_{3}+M}] \end{array}$$ (14)

**Step 2–4**: Generate *N*-dimensional initial diffusion vectors (*Ⅳ*_3_, *Ⅳ*_4_) with *Ⅳ* and *Ⅴ*, as follows.


$$\begin{array}{l} IV_{3}=IV[n_{3}+Max(M,N)+1:n_{3}+Max(M,N)+N]\\ \;=[r_{n_{3}+Max(M,N)+1},\ldots ,r_{n_{3}+Max(M,N)+N}]\\ IV_{4}=V[n_{3}+Max(M,N)+1:n_{3}+Max(M,N)+N]\\ \;=[s_{n_{3}+Max(M,N)+1},\ldots ,s_{n_{3}+Max(M,N)+N}] \end{array}$$ (15)

### Step 3: Global rewriting

The image matrix **I** is rewritten globally by the random row-rewriting vector *Ⅳ*_1_ and column-rewriting vector *Ⅳ*_2_ to generate the rewriting matrix **I**_1_, as follows:

$$I_{1}={\left((((I+IV_{1})\mathrm{mod}\;{256)}^{T}+IV_{2})\mathrm{mod}\;256\right)}^{T}$$ (16)

where ‘**A**+*B*’ is to sum the elements in vector *B* and the elements in each row of matrix **A** in turn.

### Step 4: Plaintext-related vector diffusion with the key matrix K

**Step 4–1**: Generate the plaintext-sensitive initial row diffusion position *P*_*r*_ and column diffusion position *P*_*c*_ with the statistical value of the image **I**, as follows:

$$\begin{array}{l} P_{r}=(\sum_{i=1}^{M}\sum_{j=1}^{N}I[i][j])\mathrm{mod}M\\ P_{c}=(\sum_{j=1}^{N}I[P_{r}][j])\mathrm{mod}N \end{array}$$ (17)

**Step 4–2**: Perform the first round of row diffusion on the rewriting matrix **I**_1_ with plaintext-related initial position *P*_*r*_ and random initial diffusion vectors (*Ⅳ*_3_, *Ⅳ*_4_) to generate the matrix **TC**_1_. This process involves the parallel operation of two vectors, as follows (where A[*k*,:] represents the row *k* of the matrix **A**):

{TC1[2i,:]=(temp1⊕I1[(2×(i+Pr))modM,:])mod256TC1[2i+1,:]=(temp2⊕I1[(2×(i+Pr))modM+1,:])mod2560≤i≤M2−1{temp1=(K[2i,:]+IV3)mod256temp2=(K[2i+1,:]+IV4)mod256ifi=0{temp1=(K[2i,:]+TC1[2(i−1),:])mod256temp2=(K[2i+1,:]+TC1[2i−1,:])mod256if1≤i≤M2−1 (18)

**Step 4–3**: Perform the second round of row diffusion on the matrix **TC**_1_ to generate the matrix **TC**_2_, as follows:

{TC2[2i,:]=(temp1⊕TC1[2i,:])mod256TC2[2i+1,:]=(temp2⊕TC1[2i+1,:])mod2560≤i≤M2−1{temp1=(K[2i,:]+TC1[M−1,:])mod256temp2=(K[2i+1,:]+TC1[M−2,:])mod256ifi=0{temp1=(K[2i,:]+TC2[2(i−1),:])mod256temp2=(K[2i+1,:]+TC2[2i−1,:])mod256if1≤i≤M2−1 (19)

**Step 4–4**: Perform the first round of column diffusion on the matrix **TC**_2_ to generate the matrix **TC**_3_, as follows (where A[:,*k*] represents the column *k* of the matrix **A**):

{TC3[:,2i]=(temp1⊕TC2[:,2×(i+Pc)modN])mod256TC3[:,2i+1]=(temp2⊕TC2[:,2×(i+Pc)modN+1])mod2560≤i≤N2−1{temp1=(K[:,2i]+TC2[:,N−1])mod256temp2=(K[:,2i+1]+TC2[:,N−2])mod256ifi=0{temp1=(K[:,2i]+TC3[:,2(i−1)])mod256temp2=(K[:,2i+1]+TC3[:,2i−1])mod256if1≤i≤N2−1 (20)

**Step 4–5**: Perform the second round of column diffusion on the matrix **TC**_3_ to generate the cipher matrix **E**, as follows:

{E[:,2i]=(temp1⊕TC3[:,2i])mod256E[:,2i+1]=(temp2⊕TC3[:,2i+1])mod2560≤i≤N2−1{temp1=(K[:,2i]+TC3[:,N−1])mod256temp2=(K[:,2i+1]+TC3[:,N−2])mod256ifi=0{temp1=(K[:,2i]+C[:,2(i−1)])mod256temp2=(K[:,2i+1]+C[:,2i−1])mod256if1≤i≤N2−1 (21)

## 6. Image partition security-sharing process

**Fig 8:**
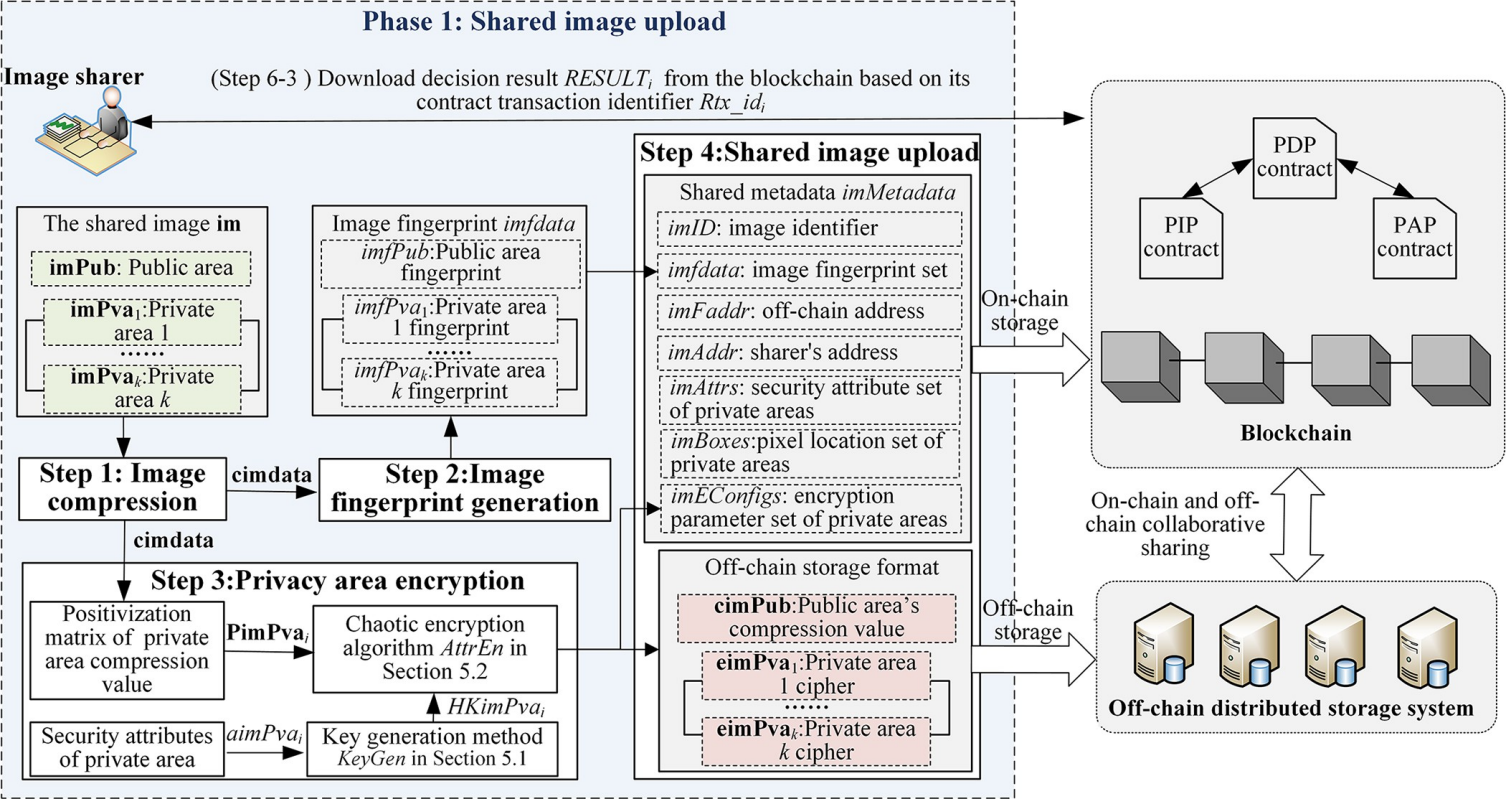
The upload process of shared image.

**Fig 9:**
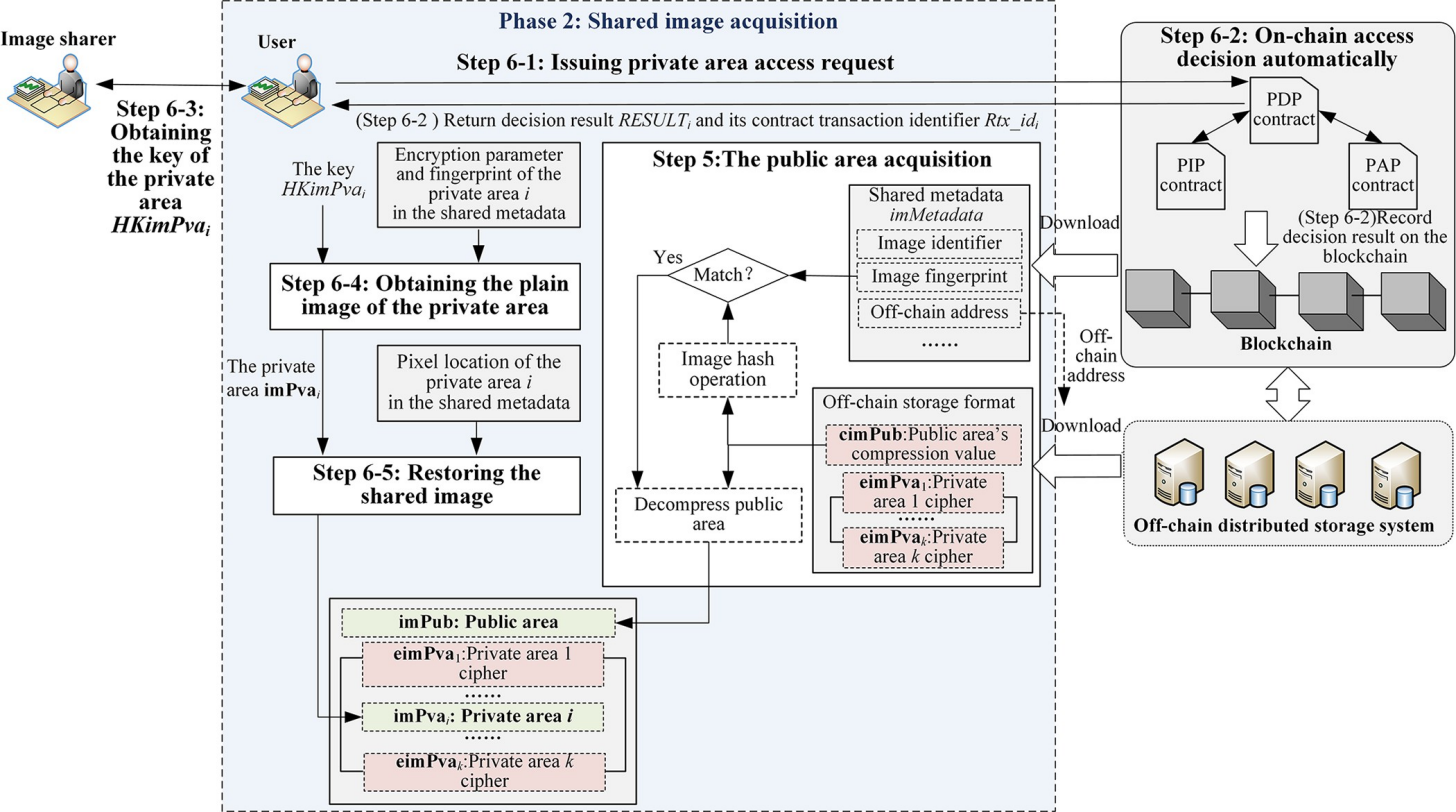
The acquisition process of shared image.

The following is a detailed description of the image partition security-sharing process, which is divided into two phases: shared image upload (as shown in Fig 8) and shared image acquisition (as shown in Fig 9).

### (1) Phase 1: Shared image upload

**Step 1: (Image compression)** The image sharer compresses the public area **imPub** and the *k* private areas {**imPva**_1_,…,**imPva**_*k*_} of the shared image **im** by using the image compression algorithm to generate the image compression data **cimdata** = {**cimPub,cimPva**_1_,…,**cimPva**_*k*_}.

Image compression can reduce storage overhead and transmission bandwidth. Additionally, image compression before encryption can decrease data redundancy and increase the difficulty of cryptanalysis.

**Step 2: (Image fingerprint generation)** The image sharer respectively calculates the hash values of the public area compression value **cimPub** and the private area compression values {**cimPub,cimPva**_1_,…,**cimPva**_*k*_} to generate the image fingerprint *imfdata* = {*imfPub*, *imfPva*_1_,…, *imfPva*_*k*_}.

**Step 3: (Private areas encryption)** The image sharer encrypts the private area compression values {**cimPva**_1_,…,**cimPva**_*k*_} with their respective keys {*HKimPva*_1_,…,*HKimPva*_*k*_} to generate the cipher images {**eimPva**_1_,…,**eimPva**_*k*_}, as follows:

$$\begin{array}{l} HKimPva_{i}=KeyGen(aimPva_{i})\\ eimPva_{i}=AttrEn(HKimPva_{i},PimPva_{i}) \end{array}$$ (22)

where *aimPva*_*i*_ is the security attribute set of the private area **imPva**_*i*_; **PimPva**_*i*_ is the positivization matrix of the matrix **cimPva**_*i*_, 1≤*i*≤*k*; *KeyGen*(•) is the security attribute-related key generation method proposed in Section 5.1; *AttrEn*(•) is the chaotic encryption algorithm proposed in Section 5.2.

To ensure the modular operation of chaotic encryption is not affected by negative numbers in the image compression value, a positivization operation is introduced before encryption. This eliminates any numerical differences, as shown in Formula (23).

$$PimPva_{i}=cimPva_{i}⊙FimPva_{i}$$ (23)

where **cimPva**_*i*_ is the compression value of the private area **imPva**_*i*_, and it can be represented as a matrix [*s*_*jk*_]_*m*×*n*_; **FimPva**_*i*_ = [*f*_*jk*_]_*m*×*n*_, and $f_{jk}=\{\begin{array}{cc} 1, & s_{jk}>0\\ -1, & s_{jk}<0 \end{array}$; ⊙ represents bitwise multiplication, 1≤*i*≤*k*.

The encryption parameter set $imEConfigs=\{imEConfig_{1},\ldots ,imEConfig_{k}\}$ is constructed, and $imEConfig_{i}=(FimPva_{i},P_{r}^{i},P_{c}^{i})$, where $P_{r}^{i}$ is the initial row diffusion position, and $P_{c}^{i}$ is the initial column diffusion position, 1≤*i*≤*k*.

**Step 4: (Shared image upload)** The image sharer uploads the off-chain storage format {**cimPub**,**eimPva**_1_,…,**eimPva**_*k*_} of the image **im** to the off-chain distributed storage system and obtains the off-chain storage address *imFaddr*. Meanwhile, the sharer uploads the shared metadata *imMetadata* to the metadata transaction set on the blockchain.

### (2) Phase 2: Shared image acquisition

**Step 5:(The public area acquisition)** Any user can download the metadata *imMetadata* of the image **im** from the blockchain. Based on the off-chain storage address *imFaddr* in the *imMetadata*, the off-chain storage format {**cimPub**,**eimPva**_1_,…,**eimPva**_*k*_} can then be downloaded from the off-chain storage system. If the **cimPub**’s hash value is consistent with *imfPub* (*imfPub*∈*imfdata*, *imfdata*∈*imMetadata*), the public area **imPub** can be obtained by decompressing **cimPub**. Otherwise, it indicates that the shared image on the off-chain storage system has been tampered with.

### Step 6: (The private area acquisition)

**Step 6–1: (Issuing private area access request)** The user submits an access request *request* = (*uID*, *aimPva*_*i*_) to the PDP contract through the PEP client, where *uID* is the user’s unique identifier, *aimPva*_*i*_ is the security attributes of a private area, 1≤*i*≤*k*.

**Step 6–2: (On-chain Access Decision Automatically)** According to the access control process introduced in Section 4.1, the PDP contract finally generates the decision result $RESULT_{i}=\{Ptx\_id_{i},uID,aimPva_{i},ALLOW/REJECT\}$, records it in the contract transaction set on the blockchain, obtains the contract transaction identifier *Rtx_id*_*i*_, and returns *Rtx_id*_*i*_, *RESULT*_*i*_ to the user.

**Step 6–3: (Obtaining The Key of The Private Area)** If the decision result is ‘*ALLOW*’, the user sends *Rtx_id*_*i*_ to the image sharer according to his address *imAddr* in the *imMetadata*. The image sharer downloads *RESULT*_*i*_ from the blockchain based on *Rtx_id*_*i*_, retrieves the key *HKimPva*_*i*_ based on *aimPva*_*i*_ in the *RESULT*_*i*_, then encrypts *HKimPva*_*i*_ with the user’s public key, and sends the ciphertext to the user. The user decrypts with his private key to obtain the key *HKimPva*_*i*_.

**Step 6–4: (Obtaining the plain image of the private area)** The user firstly decrypts the cipher image **eimPva**_*i*_ with *HKimPva*_*i*_ and $P_{r}^{i}$, $P_{c}^{i}$ to obtain **PimPva**_*i*_ by using the chaotic decryption algorithm in S1 Appendix, then restores the compression value **cimPva**_*i*_ with **FimPva**_*i*_ (i.e. **cimPva**_*i*_ = **PimPva**_*i*_⊙**Fimpva**_*i*_), finally verifies whether the **cimPva**_*i*_ ‘s hash value is consistent with *imfPva*_*i*_ (*imfPva*_*i*_ ∈ *imfdata*, *imfdata* ∈ *imMetadata*) and decompress **cimPva**_*i*_ to obtain the private area **imPva**_*i*_ if they are consistent. Otherwise, the cipher image on the off-chain storage system is tampered with.

**Step 6–5: (Restoring the shared image)** The user restores the shared image with the public area **imPub** and the private area **imPva**_*i*_ based on the pixel position *imBox*_*i*_ of the private area in the image.

## 7. Experimental datasets and platform

### 7.1 Experimental datasets

The two image datasets are employed for security analysis and performance evaluation. The first image dataset encompasses eight standard images: Cameraman256, Cameraman512, Baboon512, Barbara512, Goldhill512, Lena512, Peppers512, and 3.2.25–1024, which is provided in S1 Data in Supporting Information. These images are open and standard images that are widely used in the field of image processing. The dataset will be employed to analyze the security and evaluate the performance of the proposed chaotic encryption scheme. Similarly, current research on chaotic encryption schemes also employs the same images for security analysis and performance evaluation. The VISPR dataset [60] is a collection of 22,000 publicly available Flickr images uploaded by users in the real world. It can be downloaded from the website: https://resources.mpi-inf.mpg.de/d2/orekondy/redactions/#dataset. The paper adheres to the VISPR dataset statement. The images in the VISPR dataset contain personal information that should be privacy-preserving, as referred to as the ‘private area’ in the paper. The dataset will be to evaluate the performance of the IPSS_BCE mechanism.

### 7.2 Experimental platform

The platform used for security analysis and performance evaluation is a personal computer running Windows 10 (64-bit) operating system and equipped with 32 GB main memory. The processor of the platform is Intel(R) Core(TM) i9-9980H@2.30 GHz, and the GPU is NVIDIA GeForce RTX 2080.

## 8. Security analysis

### 8.1 Security analysis of the proposed chaotic encryption scheme

#### 8.1.1 Statistical characteristic analysis of cipher image

**Fig 10:**
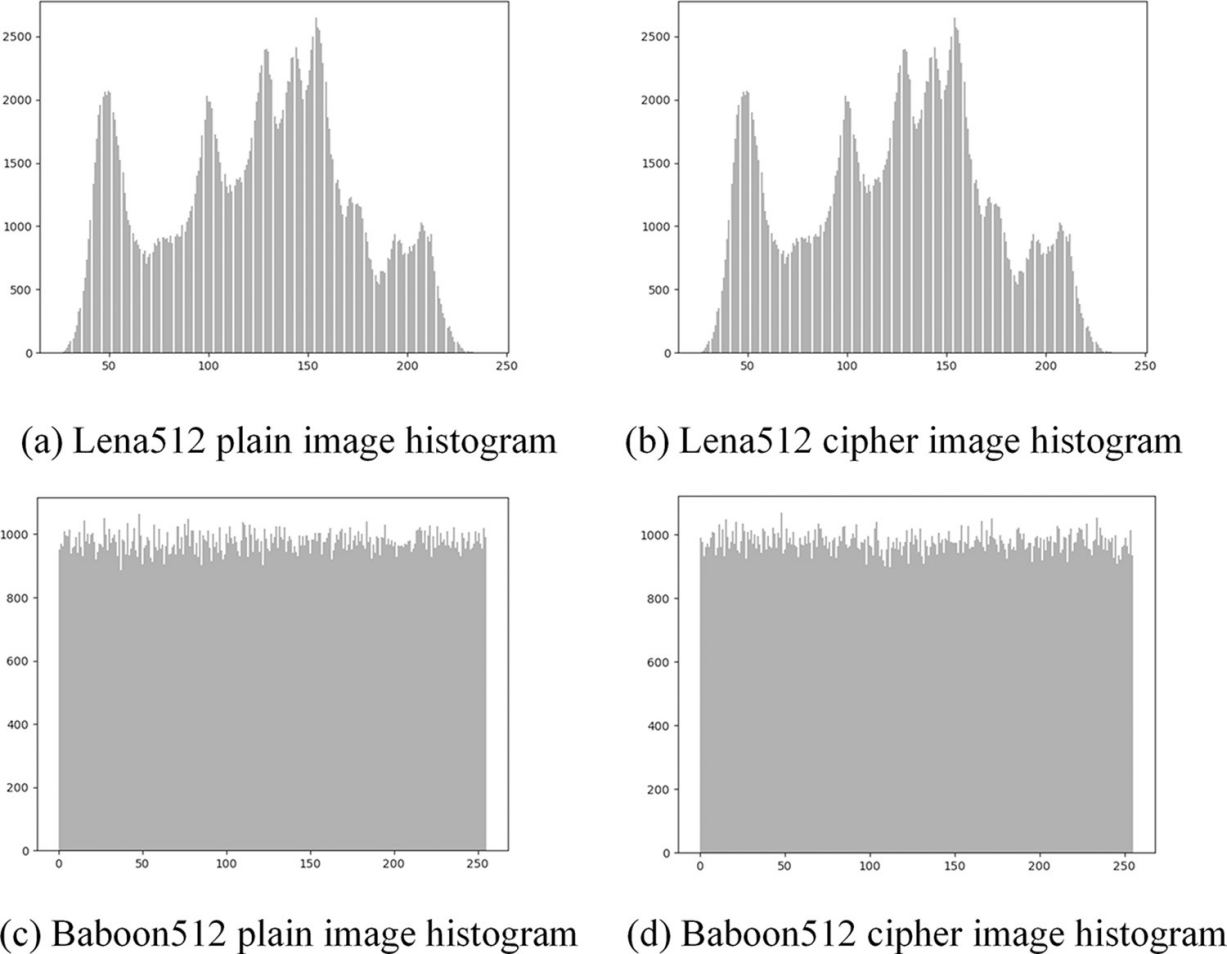
The histogram of image pixel distribution.

*(1) Histogram statistical analysis*. The image histogram intuitively reflects the distributions of pixel values in plain and cipher images. The results of the histogram analysis are presented in Fig 10. It can be seen that the histograms of plain images are unevenly distributed and have rich image statistical information, but the histograms of cipher images are almost uniformly distributed. This indicates that the proposed encryption scheme effectively hides the statistical feature information of the original plain image, making it resistant to statistical analysis attacks.

*(2) Correlation analysis of adjacent pixels*. To resist statistical attacks, the strong correlation between adjacent pixels in the plain image should be eliminated by the image encryption algorithm. In this paper, the correlation coefficient *γ* is used to evaluate the correlation between adjacent pixels in the image, and its calculation is shown in eq (30) (*a*_*i*_,*b*_*i*_ represents the value of adjacent pixels):

$$\begin{array}{l} \gamma =\frac{\sum_{i=1}^{N}\left(a_{i}-\bar{a})(b_{i}-\bar{b}\right)}{\sqrt{\left(\sum_{i=1}^{N}{\left(a_{i}-\bar{a}\right)}^{2})(\sum_{i=1}^{N}{\left(b_{i}-\bar{b}\right)}^{2}\right)}}\\ \bar{a}=\frac{1}{N}\sum_{i=1}^{N}a_{i},\bar{b}=\frac{1}{N}\sum_{i=1}^{N}b_{i} \end{array}$$ (24)

*γ*>0.6 indicates that the correlation between adjacent pixels is very strong, and *γ*≈0 indicates that the correlation between adjacent pixels is broken. To ensure the reliability of the experiment, 1000 groups of adjacent pixels in the horizontal, vertical, and diagonal directions are randomly selected to calculate the correlation coefficient. The results are presented in Table 2. It can be seen that the correlation coefficients of the cipher images are significantly reduced and close to 0, indicating that the proposed encryption scheme can effectively break the strong correlation of adjacent pixels in the plain image.

**Table 2:** The correlation analysis results of adjacent pixels.

Image	Horizontal direction		Vertical direction		Diagonal direction	
	Plain image	Cipher image	Plain image	Cipher image	Plain image	Cipher image
**Lena512**	0.9271	-0.0045	0.9657	-0.0075	0.9234	0.0056
**Baboon512**	0.9452	-0.0053	0.9341	0.0031	0.9241	-0.0048

*(3) Chi-square analysis*. The Chi-square test is an important method to quantitatively analyze the uniform distribution of pixel values in cipher images. The Chi-square value of a cipher image is calculated as follows:

$$\gamma^{2}=\sum_{i=1}^{K}\frac{\left(n_{i}-M\times N\times p\right)}{M\times N\times p}$$ (25)

where *K* is the number of pixel types in the image, and *K* = 256 for gray images, *n*_*i*_ is the number of pixel type *i* in the image, *M* and *N* are the row and column values of the image, *p* = 1/*K*.

When the significance level *a* = 0.05, the theoretical value of the Chi-square test is 293.2478. If the Chi-square value of the cipher image is smaller than the theoretical value, the cipher image passes the Chi-square test. As shown in Table 3, the Chi-square values of the cipher images encrypted by the proposed encryption algorithm are smaller than the theoretical value. Thus, the proposed encryption scheme passes the Chi-square test and can well resist statistical analysis attacks.

**Table 3:** Chi-square analysis.

Image	*γ* ^2^	Result
**Lena512**	263.1895	**pass**
**Baboon512**	256.6707	**pass**
**Cameraman256**	270.2623	**pass**
**Barbara512**	262.9007	**pass**
**Goldhill512**	243.4222	**pass**
**Peppers512**	262.4932	**pass**

#### 8.1.2 Information entropy analysis

Information entropy is an important indicator for measuring the randomness of the signal source, and it is calculated as follows [61]:

$$H(m)=-\sum_{i=0}^{2^{K}-1}p(g_{i})\log_{2}p(g_{i})$$ (26)

where *K* is the number of bits to represent a symbol *g*_*i*_, 2^*K*^ represents the total states of *g*_*i*_, and *p*(*g*_*i*_) is the occurrence probability of *g*_*i*_.

An 8-bit truly random gray image (*K* = 8) has a uniform distribution of pixel intensities in the interval [0, 255]. According to Eq (26), the information entropy of an 8-bit gray image is 8 in the ideal case. As shown in Table 4, the information entropy values of the cipher images encrypted by the proposed scheme are close to the ideal value of 8, reaching more than 7.999. This indicates that these cipher images are highly random, and that the proposed scheme has a good encryption effect. The proposed scheme is comparable to the image encryption scheme in reference [59], and superior to references [39, 40] in terms of information entropy.

**Table 4:** Information entropy analysis. (‘CR’ represents the image compression ratio).

Image	[59]	[40] (CR = 1)	[39]	Proposed scheme
**Lena512**	7.9993	7.9988	7.9974	**7.9994**
**Baboon512**	7.9996	7.9994	7.9972	**7.9995**
**Cameraman256**	7.9995	7.9967	7.9970	**7.9996**

#### 8.1.3 Key analysis

*(1) Key space analysis*. The proposed chaotic encryption scheme has a key length of 256 bits, and its key space is 2^256^, which is much larger than the required value 2^100^ [62]. Therefore, the proposed chaotic encryption scheme can resist brute-force attacks.

*(2) Key sensitivity analysis*. Key sensitivity refers to the phenomenon that a slight change to the key produces a completely different cipher image. The stronger the key sensitivity of the encryption scheme, the higher the security of encryption. In this paper, the Number of Pixels Change Rate (NPCR) and the Unified Average Change Intensity (UACI) parameters are used to evaluate the key sensitivity of the chaotic encryption scheme, which respectively represent the proportion and average change intensity of the number of pixel changes between two cipher images. The calculation formulas are shown in Eq (27). The ideal NPCR and UACI values of gray images are 99.61% and 33.46% [63], respectively.

$$\begin{array}{l} \mathrm{NPCR}=\frac{1}{M\times N}\sum_{i=1}^{M}\sum_{j=1}^{N}D(i,j)\times 100\%\\ D(i,j)=\{\begin{array}{cc} 1 & C_{1}(i,j)=C_{2}(i,j)\\ 0 & C_{1}(i,j)\neq C_{2}(i,j) \end{array}\\ \mathrm{UACI}=\frac{1}{M\times N}\sum_{i=1}^{M}\sum_{j=1}^{N}\frac{\left|C_{1}(i,j)-C_{2}(i,j)\right|}{255}\times 100\% \end{array}$$ (27)

where *C*_1_(*i*,*j*) is the pixel at the location (*i*,*j*) in the cipher image with the original key, *C*_2_(*i*,*j*) is the pixel at the corresponding location (*i*,*j*) in the cipher image with a slightly changed key, *M* and *N* are the row and column values of the image.

According to the initial value and pre-iteration value calculation method in the proposed encryption scheme, the key ‘a06ff1db30975a222c2d664a4e866fc02677275 faaa6b164a0d28b2d45a6ae8’ is divided into nine segments, and the lowest bit of each segment is flipped. Table 5 shows the NPCR and UACI values obtained by encrypting the image Lena512 with these keys. The results demonstrate that a one-bit change in the key produces a completely different cipher image, highlighting the excellent key sensitivity of the proposed encryption scheme.

**Table 5:** Key sensitivity analysis.

Initial key segment	Changed key segment	Proposed scheme	
		NPCR (%)	UACI (%)
A06ff1db30	A06ff1db31	99.61	33.54
9759a222c2	9759a222c3	99.61	33.49
D664a4e866	D664a4e867	99.61	33.48
Fc02677275	Fc02677276	99.60	33.54
Faaa6b164a	Faaa6b164b	99.64	33.43
0d	0C	99.63	33.49
28b2d	28b2c	99.61	33.45
A06ff1db30	A06ff1db31	99.61	33.54
9759a222c2	9759a222c3	99.61	33.49

#### 8.1.4 Anti-attack ability analysis

*(1) Anti-differential attack analysis*. To resist differential attack [43], a slight change to the plain image should be uniformly diffused to the whole cipher image by the chaotic encryption scheme. The NPCR and UACI parameters are also used to evaluate the plaintext sensitivity of the chaotic encryption scheme. In Eq (27), *C*_1_(*i*,*j*) and *C*_2_(*i*,*j*) are the pixels at the location (*i*,*j*) in the cipher image of the original plain image and the slightly changed plain image, respectively.

In the experiment, the lowest bit of the pixel value at a specific position in the plain image is flipped. Table 6 shows the results of 100 experiments. It is evident that the proposed encryption scheme has good plaintext sensitivity and a strong ability to resist differential attacks, as the NPCR and UACI values are closer to the ideal values.

**Table 6:** Anti-differential attack analysis.

Image	[59]		[40]		[39]		Proposed scheme	
	NPCR(%)	UACI(%)	NPCR(%)	UACI(%)	NPCR(%)	UACI(%)	NPCR(%)	UACI(%)
**Lena512**	99.61	33.46	99.61	33.46	99.88	33.34	**99.61**	**33.46**
**Baboon512**	99.61	33.47	99.61	33.46	99.87	33.34	**99.61**	**33.47**
**Cameraman256**	99.61	33.46	99.61	33.48	99.88	33.35	**99.61**	**33.46**

It should be noted that the encryption scheme proposed in this paper does not use the plaintext-related key generation method, which is commonly employed by most current image encryption schemes. Instead, the scheme calculates the initial row and column diffusion positions with the statistical value of the plain image. Thus, slight changes to the plain image will result in corresponding changes to the initial row and column diffusion positions, ultimately producing a completely different cipher image.

**Fig 11:**
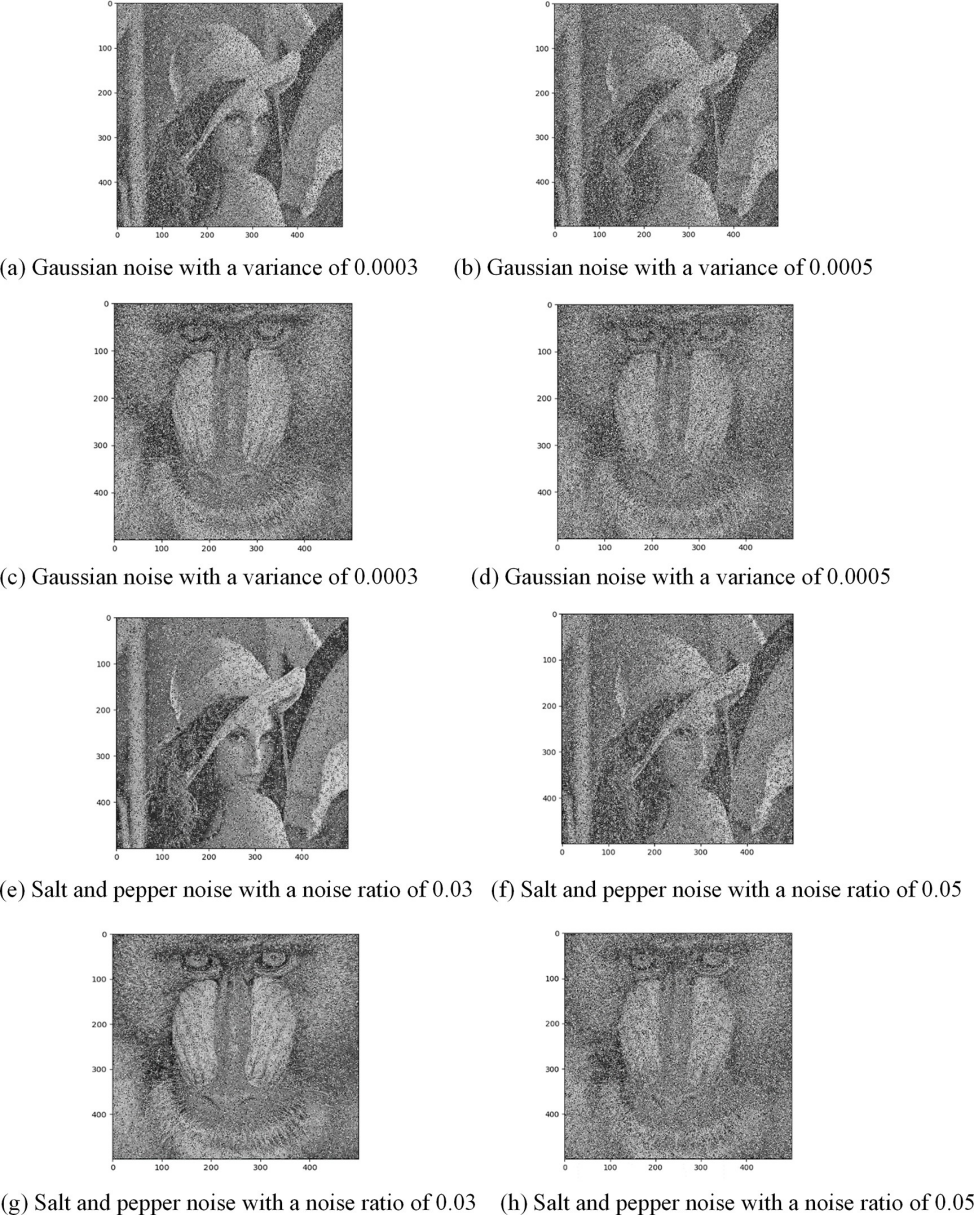
The decrypted image under Gaussian noises and salt and pepper noises.

*(2) Anti-salt and pepper*, *gaussian noise attack analysis*. The cipher image may be affected by various communication noises during transmission. If the cipher image is corrupted by noise, the encryption scheme should be able to restore the original plain image. The experiment involved adding Gaussian noises with a variance of 0.0003 and 0.0005, and salt and pepper noises with a noise ratio of 0.03 and 0.05 to the cipher image. The cipher images polluted by these noises are then decrypted, and the decrypted images are illustrated in Fig 11. It can be seen that the proposed encryption scheme in this paper can successfully restore the original plain image from the cipher image polluted by Gaussian noises and salt and pepper noises. Thus, the proposed encryption scheme has strong robustness and can meet the requirement of image transmission in the open channel.

### 8.2 Security analysis of the proposed IPSS_BCE mechanism

To achieve secure sharing of images by partition, private areas in the shared image should be encrypted and can only be decrypted and accessed by users who meet the policy requirements.

The IPSS_BCE mechanism uses the proposed security attribute-related chaotic encryption scheme to encrypt private areas in the shared image. According to the security analysis results in Section 8.1, the proposed encryption scheme has a good image encryption effect. The security of key distribution is analyzed below. After a user requests the key of a private area from the image sharer, the image sharer obtains the decision result (*RESULT*) from the blockchain based on its contract transaction identifier (*Rtx_id*). The tamper-resistant nature of the blockchain guarantees the authenticity of the result (*ALLOW/REJECT*), the user’s identifier (*uID*), and the private area’s security attribute (*aimPva*) in the decision result (*RESULT*). The image sharer retrieves the key based on the private area’s security attribute (*aimPva*) and encrypts it with the public key of the user with *uID* to ensure confidentiality during distribution. The cipher key can only be decrypted by the user with *uID*. If the requester of the key is fraudulent, they will be unable to decrypt the key correctly, ensuring the security of key distribution.

The IPSS_BCE mechanism adopts the ABAC model. Hence, the access control policy of the private area is defined based on the security attributes of the user and the private area. All access control policies and user security attributes are stored on the blockchain to ensure that these policies and attributes cannot be tampered with. The PDP contract then makes access decisions based on these authentic policies and attributes. Meanwhile, smart contracts can achieve reliable and automatic decisions on user’s access requests. The decision result is recorded in the contract transaction set of the blockchain to ensure transparency and non-tampering. Thus, to access a private area with certain security attributes, a user’s security attribute must align with the subject’s security attribute in the access control policy that restricts access to this private area. Based on this, the user can obtain the key of the private area, decrypt it, and restore the private area.

## 9. Performance evaluation

### 9.1 Performance evaluation of the proposed chaotic encryption scheme

#### 9.1.1 Time complexity

Due to adopting vector operation acceleration, the proposed chaotic encryption scheme has low time complexity. The time complexity of the global rewriting operation in the proposed scheme is *O*(*M*+*N*). The time complexity of the vector diffusion in the proposed scheme is also *O*(*M*+*N*), since the time complexity of one round of row diffusion and column diffusion is M2 and N2, respectively. So, the total time complexity of the proposed scheme can be approximated to *O*(*M*+*N*). The proposed scheme has equivalent time complexity to the chaotic encryption algorithm based on vector-level diffusion [59], and it is superior to the other chaotic encryption algorithms [39, 40] with the time complexity of *O*(*MN*). The encryption time evaluation results are shown in Table 7.

**Table 7:** Time complexity analysis.

Image	[59](s)	[40](s)	[39](s)	Proposed scheme(s)
**Cameraman256**	0.0126	0.0653	0.1189	**0.0063**
**Lena512**	0.0248	0.2676	0.4452	**0.0166**
**3.2.25–1024**	0.0607	1.0964	1.6673	**0.0502**

#### 9.1.2 The number of keys

This section analyzes the number of keys required by the chaotic encryption scheme proposed in this paper. Suppose that the size of the shared image dataset is *n*, the number of private areas in each image is *num*_*i*_ (1≤*i*≤*n*), and the total number of security attributes for private areas is *num*_*ATTR*_. For the proposed ‘one attribute, one key’ encryption scheme, the total number of keys is *N*_*T*_ = *num*_*ATTR*_; for current ‘one image, one key’ encryption schemes, the total number of keys is $N_{T}^{\prime }=\sum_{i=1}^{n}num_{i}$ due to the use of different keys for each private area. Generally, the security attribute of an image describes common features of a class of images based on security requirements, so $N_{T}\ll n<N_{T}^{\prime }$. Taking the VISPR real dataset [60] as an example, it contains 28 types of security attributes related to user privacy (such as ‘face’, ‘ID card number’, etc.) and 48846 private areas. That is, *N*_*T*_ = 28, and $N_{T}^{\prime }$ = 48846. It can be seen that the security attribute-related key generation method proposed in this paper significantly reduces the number of keys and the burden of key management.

### 9.2 Performance evaluation of the proposed IPSS_BCE mechanism

#### 9.2.1 Retrieval efficiency

The efficiency of the proposed on-chain transaction retrieval method based on the Cuckoo filter is evaluated on the Hyperledger Fabric v1.4 data structure and compared with the retrieval method based on the Bloom filter. In the evaluation, each block stores 50 transactions. For the Cuckoo filter, there are 50 buckets (*n* = 50), each containing 8 entries (*b* = 8), and the fingerprint length of the key value is 16 (*l* = 16). According to Eq (28), the false-positive rate of the Cuckoo filter is 0.39% (*ε* = 0.39%).


$$\varepsilon =1-{\left(1-1/2^{l}\right)}^{2b}\approx 2b/2^{l}$$ (28)

For the Bloom filter, under the same false-positive rate *ε* and the element number *n* as those in the Cuckoo filter, the length of bit array is 128 bit (*p* = 128) and the number of hash functions is 2 (*k* = 2), according to the calculation methods in Eq (29).


$$\begin{array}{l} p=-\frac{n\ln\varepsilon }{{\left(\ln2\right)}^{2}}\\ k=\frac{p}{n}\ln2 \end{array}$$ (29)

**Fig 12:**
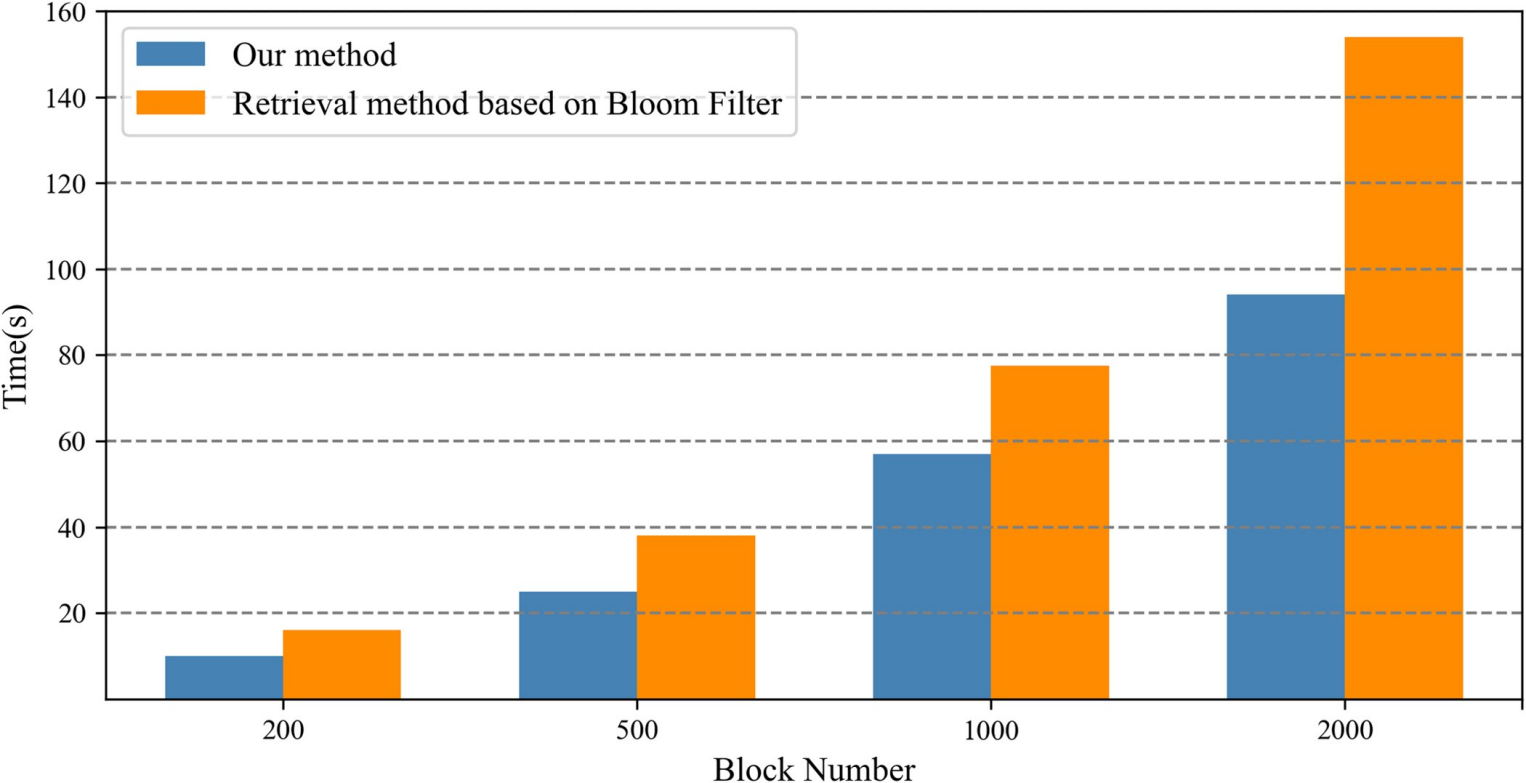
Evaluation of retrieval efficiency.

Experiments were conducted on 200, 500, 1000, and 2000 blocks, respectively. The experimental results are shown in Fig 12. The proposed retrieval method based on the Cuckoo filter has higher retrieval efficiency than that based on the Bloom filter.

#### 9.2.2 The image upload and download time overhead

The section evaluates the image upload and download time overhead by comparing the IPSS_BCE mechanism with the mechanisms outlined in reference [30, 33] based on the number of shared images. In the experiments, each image is 2 MB in size and includes a private area of 500×500 pixels. Both the IPSS_BCE mechanism and the mechanism outlined in reference [30] employ the RSA algorithm to encrypt the image encryption key. IPFS is initialized using go-ipfs [64] and interacts with the IPFS network from a local computer. In the IPSS_BCE mechanism, the private area of each image is encrypted by the proposed chaotic encryption algorithm, while the entire image is encrypted by the AES algorithm in the mechanisms referenced in [30, 33].

**Fig 13:**
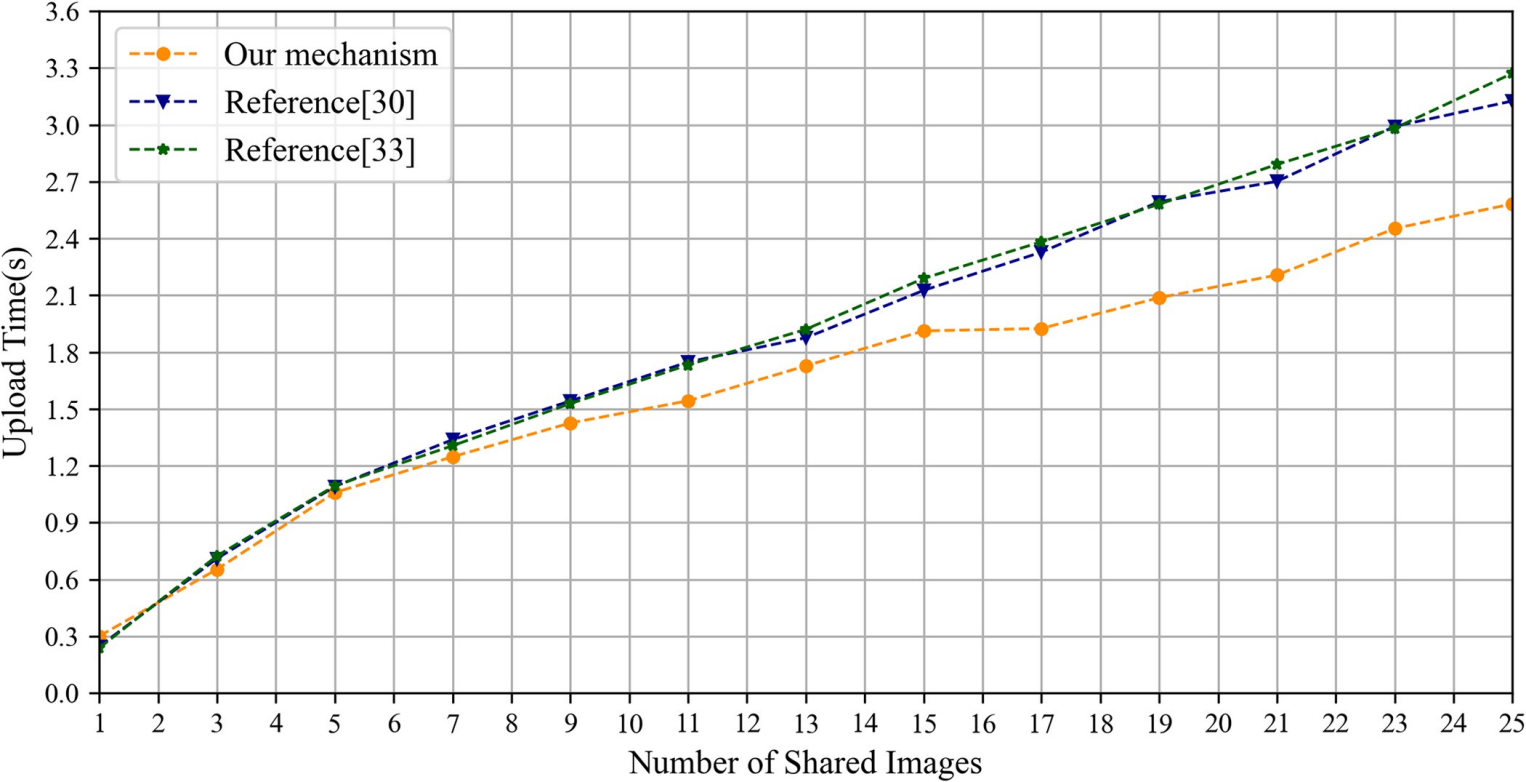
The upload time of shared images.

**Fig 14:**
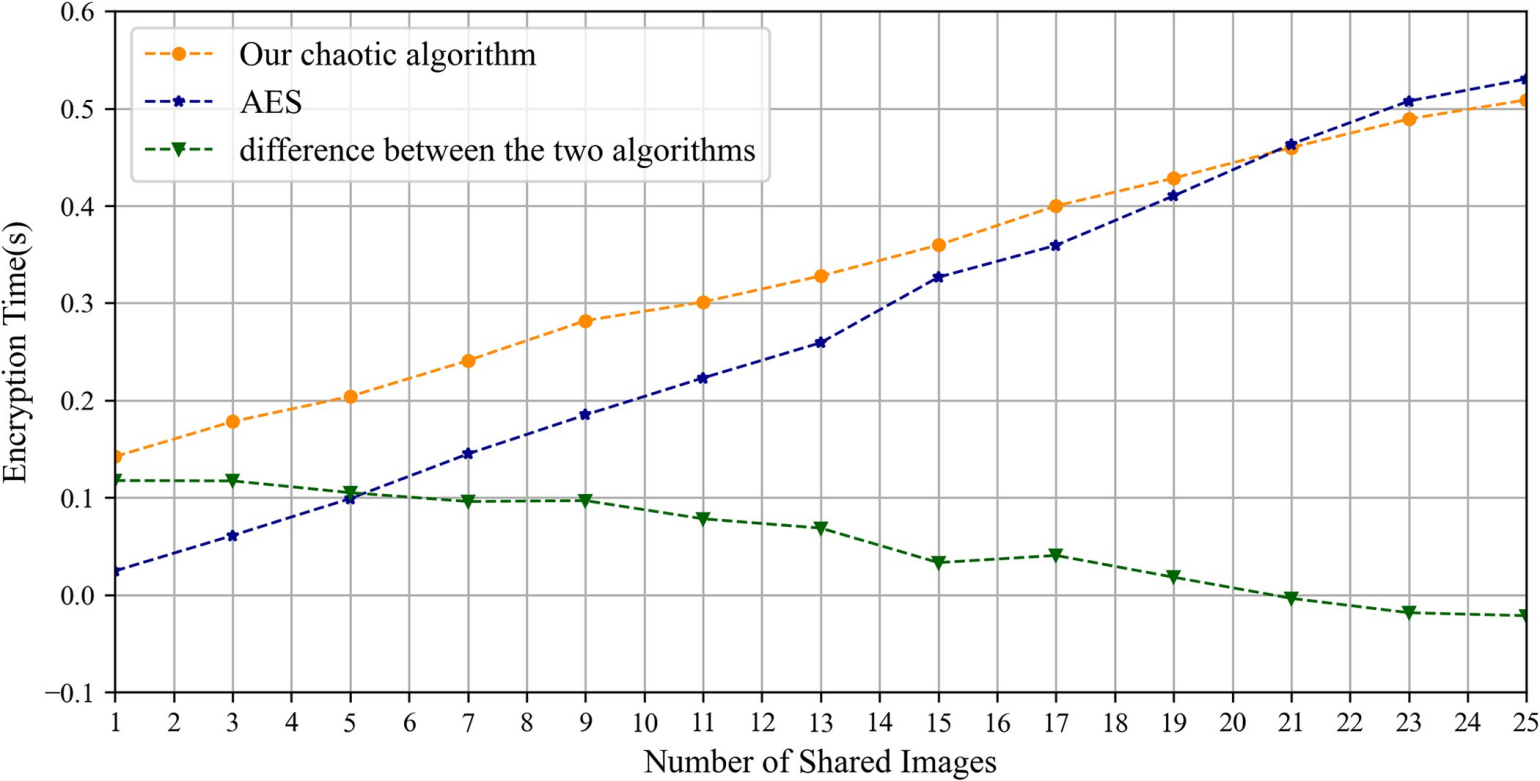
The encryption time of the proposed chaotic algorithm and AES.

**Fig 15:**
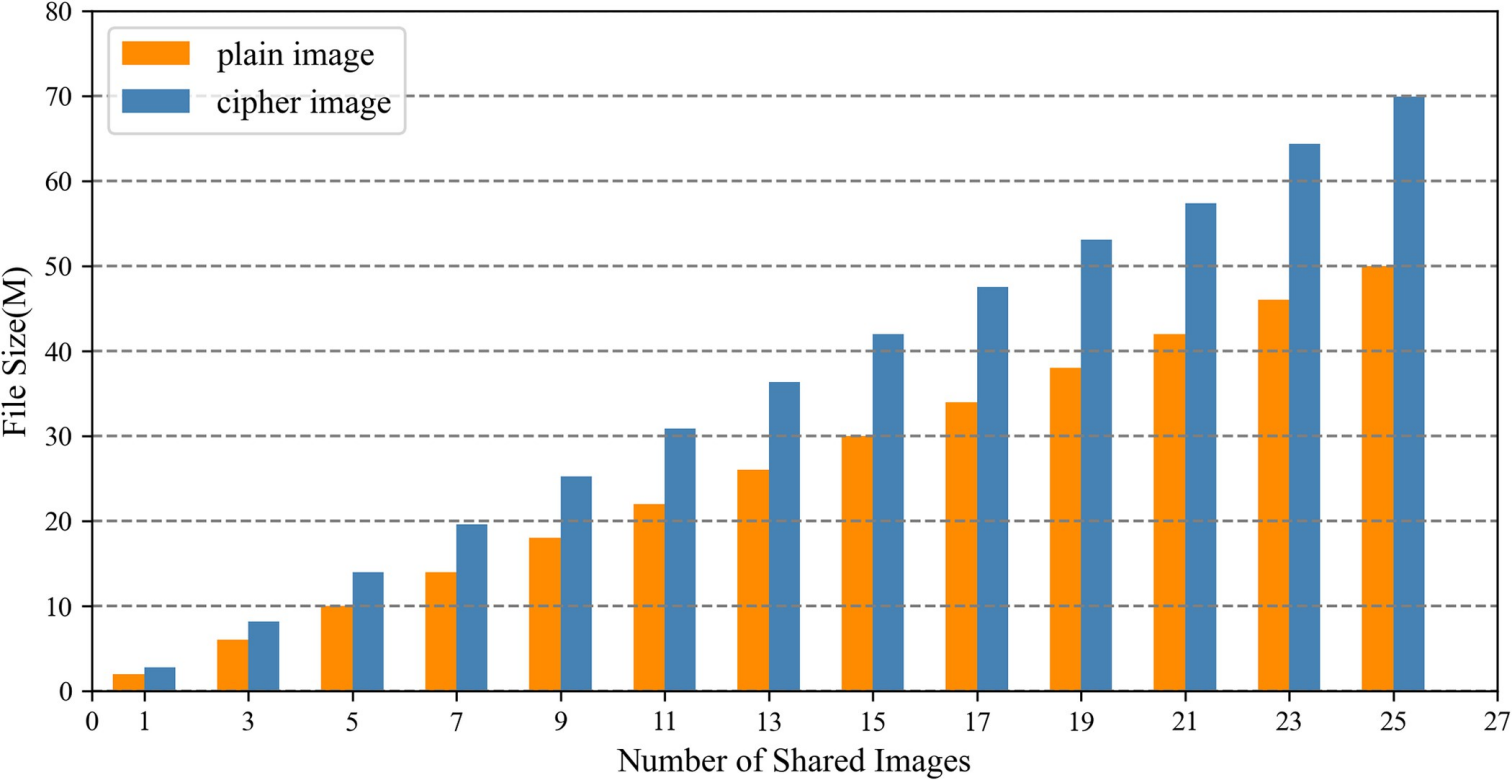
The file sizes of AES plain image and cipher image.

The experimental results of the upload time are shown in Fig 13. Notably, as the number of shared images increases, the upload time of the IPSS_BCE mechanism progressively outperforms that of the mechanisms outlined in reference [30, 33]. It is primarily affected by the image encryption time and the duration required to write the encrypted image onto IPFS. First, the encryption time of the proposed chaotic algorithm depends on the size of the private area, whereas the encryption time of the AES algorithm corresponds to the file size of the plain image. Thus, as the number of shared images grows, the encryption time of the proposed chaotic algorithm gradually surpasses that of the AES algorithm, as shown in Fig 14. Second, the duration required to write the cipher image onto IPFS depends largely on the file size being written. The file sizes of the ciphertext and plaintext of our chaotic encryption algorithm remain identical, while the file size of the AES algorithm’s ciphertext is approximately 1.4 times larger than its plaintext, as illustrated in Fig 15. Consequently, the writing time for cipher images using the proposed chaotic algorithm is consistently faster than that for images encrypted with the AES algorithm.

**Fig 16:**
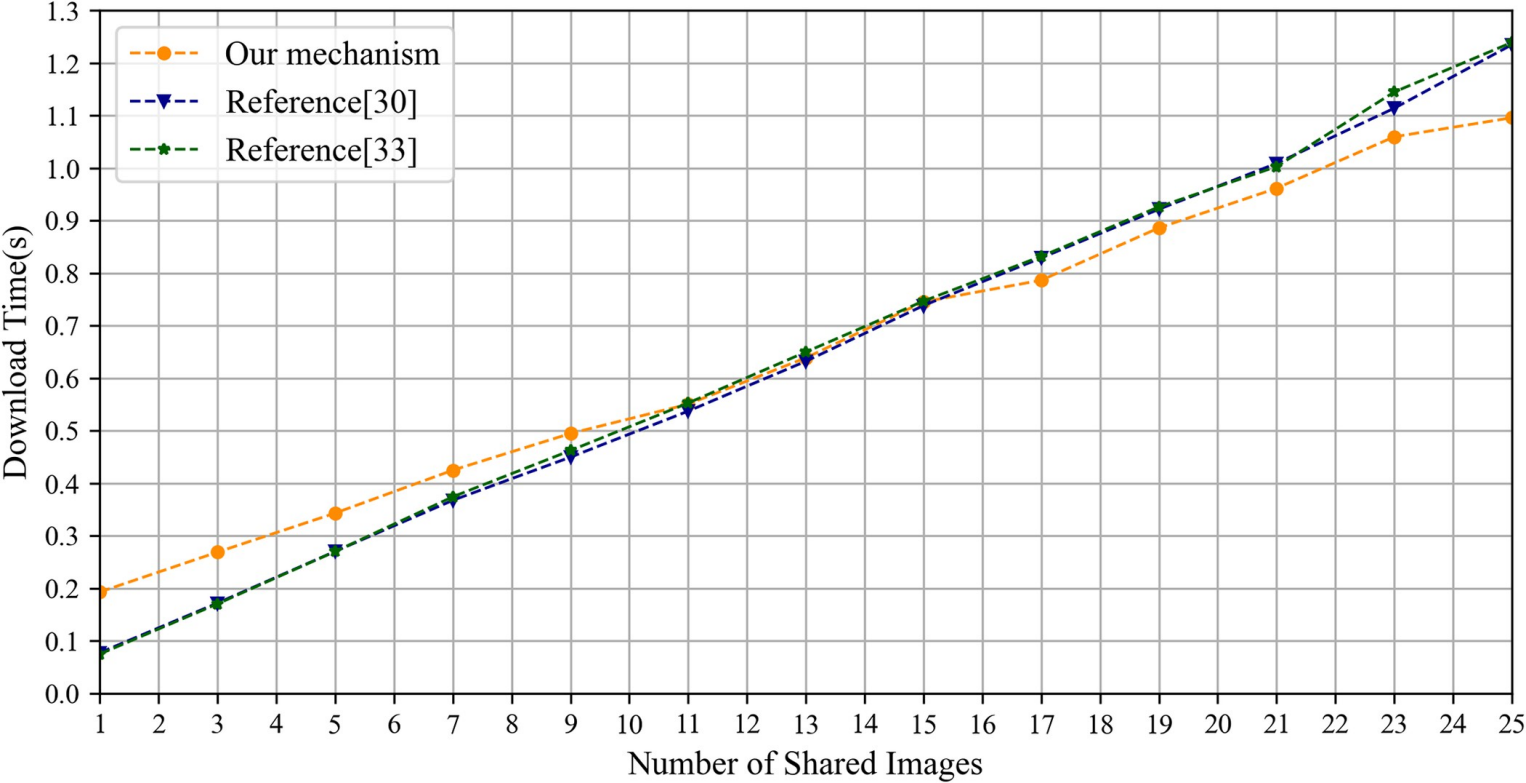
The download time of shared images.

**Fig 17:**
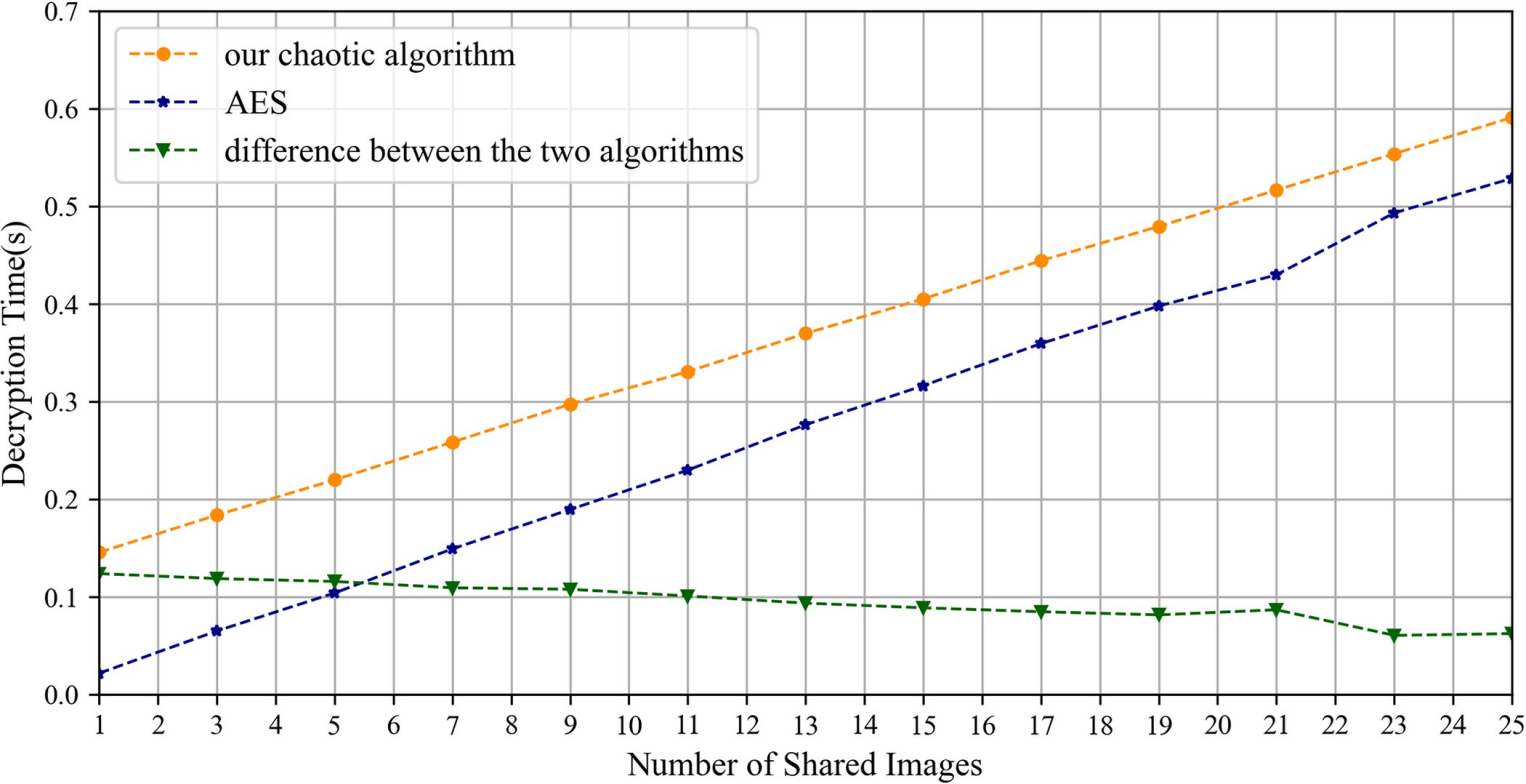
The decryption time of the proposed chaotic algorithm and AES.

Fig 16 illustrates the download time. It is evident that with the increase in the number of shared images, the download time of shared images under the IPSS_BCE mechanism is incrementally lower than that in reference [30, 33]. This is primarily attributed to the reading time of the cipher image from the IPFS and the decryption time. The reading time from the IPFS is dependent on the file size. The proposed chaotic encryption algorithm produces a cipher image that is the same size as the original plain image, while the ciphertext produced by the AES algorithm is about 1.4 times larger than its plaintext. Therefore, the reading time of the cipher image produced by the proposed chaotic encryption algorithm is inherently less than that of the AES algorithm. Meanwhile, the decryption time of the cipher image produced by the proposed chaotic encryption algorithm is gradually converging with that of the AES algorithm, as depicted in Fig 17.

## 10. Comparison with related works

Upon analysis of current research, it is observed that the most comprehensive and representative methods in the current state-of-the-art research are those referenced in [30, 33], as shown in Table 1. The proposed IPSS_BCE mechanism is compared with these methods in 12 aspects, including off-chain storage systems, access control methods, control basis and granularity, image encryption methods, and more. The comparison results are presented in Table 8. The IPSS_BCE mechanism accomplishes fine-grained, controlled image sharing at the area level, based on the security attributes of the user and private areas. This is distinct from the image-level sharing in current works. Additionally, the IPSS_BCE mechanism achieves image-specific encryption by designing a security attribute-related chaotic encryption scheme, which differs from common cryptographic algorithms used in current works. It also significantly reduces the number of image encryption keys, thus decreasing the key management burden compared to current research. Furthermore, the retrieval efficiency of on-chain attributes and policies is improved by introducing an efficient retrieval method for on-chain transactions based on the Cuckoo filter. The proposed IPSS_BCE mechanism has a lower time overhead compared to current mechanisms as the number of shared images increases.

**Table 8:** Comparison with related works.

Ref.	[30]	[33]	Proposed mechanism
**Off-chain storage**	IPFS	IPFS	IPFS
**Access Control**	ACL	CP-ABE	**ABAC**
**Control basis**	User’s identity	User’s attributes	**User’s and private area’s attributes**
**Control granularity**	Image level	Image level	**Area level**
**Image encryption**	AES	AES	**Security attribute-related chaotic encryption scheme**
**The number of keys**	$\sum_{i=1}^{n}num_{i}$	$\sum_{i=1}^{n}num_{i}$	***num***_***ATTR***_
**Key protection**	Asymmetric encryption	CP-ABE	Asymmetric encryption
**Smart contract**	Yes	Yes	Yes
**Transaction retrieval**	**-**	**-**	**On-chain transaction efficient retrieval based on the Cuckoo filter**
**Retrieval efficiency**	**As illustrated in Fig 11, the proposed mechanism exhibits higher retrieval efficiency.**		
**Fully distributed**	Yes	No	Yes
**Image upload and download time overhead**	**As demonstrated in Figs 12 and 15, the proposed mechanism has lower time overhead as the number of shared images increases.**		

where *n* is the size of the shared image dataset, *num*_*i*_ is the number of private areas in each image, 1≤*i*≤*n*, *num*_*ATTR*_ is the total number of private areas’ security attributes, $num_{ATTR}\ll n<\sum_{i=1}^{n}num_{i}$.

## 11. Limitation and discussion

The proposed IPSS_BCE mechanism achieves controlled, area-level, and privacy-preserving image-sharing through a fine-grained access control method based on ABAC, and a security attribute-related chaotic encryption scheme. The security attribute of the private area in the image is of great importance in the context of access control and the generation of the encryption key. The proposed IPSS_BCE mechanism defaults to manual calibration of the security attributes of private areas by the image sharer. Nevertheless, the manual calibration of the security attributes of private areas is challenging to accomplish when the number of images is considerable. Consequently, it is necessary to calibrate the security attributes of private areas using artificial intelligence (AI) technology. The subsequent stage of the research process is to investigate intelligent generation techniques for image security attributes.

The proposed IPSS_BCE mechanism employs a security attribute-related chaotic encryption scheme to encrypt the private area in the image. The encryption scheme is of high security but does not apply to IoT end devices with limited energy, computing power, and memory. It is necessary to develop lightweight image encryption algorithms in future research, to facilitate the application of the image partition security-sharing mechanism presented in this paper in the field of IoT.

This paper solely addresses the issue of image partition security-sharing without consideration of contribution metrics for image sharers or the ownership confirmation of shared images. These are of great importance for the promotion of image data sharing and circulation. In the future, it will be necessary to include the contribution metrics method for image sharers as a means of allocating the benefit distribution of each participant. Furthermore, a method for confirming ownership of shared images must be introduced to protect the intellectual property rights of those who contribute images.

## 12. Conclusion

This paper proposes an image partition security-sharing mechanism based on blockchain and chaotic encryption to ensure open-sharing of public areas, and fine-grained and privacy-preserving sharing of private areas in shared images. The proposed fine-grained access control method based on ABAC achieves automatic access control to shared private areas through smart contracts. It improves the retrieval efficiency of policies and attributes on the blockchain by employing the on-chain transaction retrieval method based on the Cuckoo filter. Additionally, the proposed image-specific chaotic encryption scheme reduces the number of keys required by adopting the ‘one attribute, one key’ key generation method. Results of the security analysis and performance evaluation show that the proposed mechanism has a positive impact on image encryption and also has low time complexity.

This paper presents a novel solution for privacy-preserving image data sharing. The solution will enable secure sharing of medical image data among multiple hospitals, providing convenience for patients. Additionally, the solution will secure the sharing and circulation of image data among multiple large data centres.

## Supporting information

S1 Appendix (DOCX)

S1 Data (RAR)

S1 File (RAR)
