# Supplementary material for: An image partition security-sharing mechanism based on blockchain and chaotic encryption
Source: PLoS One. 2024 Jul 29;19(7):e0307686. doi: 10.1371/journal.pone.0307686 (PMC11285975; doi:10.1371/journal.pone.0307686)
Supplement: S1 Appendix — (DOCX) [file pone.0307686.s001.docx]

**Appendix****: Chaotic decryption algorithm**

The proposed algorithm is symmetric, so the decryption key is the same as the encryption key *HK*. The key matrix **K** is also generated by the five-dimensional multi-ring multi-wing hyperchaotic system, using *HK*[1:208] as the initial value. Similarly, using *HK*[209:256] as the initial value, the random rewriting vectors (*Ⅳ*_1_, *Ⅳ*_2_) and the initial row diffusion vectors (*Ⅳ*_3_, *Ⅳ*_4_) are generated by 2D-HSM. These generation methods are identical to those of the encryption algorithm.

The decryption process is the reverse of encryption. Firstly, the cipher image matrix **E** is restored using the key matrix **K**. The input parameters for this operation include the plaintext-related row and column diffusion positions ($P_{r}$,$P_{c}$), as well as the initial row diffusion vectors (*Ⅳ*_3_, *Ⅳ*_4_). Matrix restoration is the reverse of the plaintext-related vector diffusion in the encryption process. The detailed steps are as follows:

**Step 1**: Perform the first round of column restoration from the last two columns of the cipher image matrix **E** (i.e., column *N*-1 and column *N*-2) to restore **TD**_1_, as follows:

$\begin{matrix} \left\{\begin{matrix} \mathbf{TD}_{\mathbf{1}}[:,2i]=(\mathbf{temp}_{1}\oplus\mathbf{E}[:,2i])\mathrm{mod}256 \\ \mathbf{TD}_{\mathbf{1}}[:,2i+1]=(\mathbf{temp}_{2}\oplus\mathbf{E}[:,2i+1])\mathrm{mod}256 \end{matrix}\right.\text{ 0}\leq i\leq\frac{N}{2}-1 \\ \left\{\begin{matrix} \mathbf{temp}_{1}=(\mathbf{K}[:,2i]+\mathbf{E}[:,2(i-1)])\mathrm{mod}256 \\ \mathbf{temp}_{2}=(\mathbf{K}[:,2i+1]+\mathbf{E}[:,2i-1])\mathrm{mod}256 \end{matrix}\right.\text{ if }1\leq i\leq\frac{N}{2}-1 \\ \left\{\begin{matrix} \mathbf{temp}_{1}=(\mathbf{K}[:,2i]+\mathbf{TD}_{\mathbf{1}}[:,N-1])\mathrm{mod}256 \\ \mathbf{temp}_{2}=(\mathbf{K}[:,2i+1]+\mathbf{TD}_{\mathbf{1}}[:,N-2])\mathrm{mod}256 \end{matrix}\right.\text{ if }i=0 \end{matrix}$ (30)

**Step 2**: Perform the second round of column restoration from the last two columns of **TD**_1_ to restore **TD**_2_, as follows:

$\begin{matrix} \left\{\begin{matrix} \mathbf{TD}_{\mathbf{2}}[:,2\times(i+P_{c})]=(\mathbf{temp}_{1}\oplus\mathbf{TD}_{\mathbf{1}}[:,2i])\mathrm{mod}256 \\ \mathbf{TD}_{\mathbf{2}}[:,2\times(i+P_{c})\mathrm{mod}N+1]=(\mathbf{temp}_{2}\oplus\mathbf{TD}_{\mathbf{1}}[:,2i+1])\mathrm{mod}256 \end{matrix}\right.\text{ 0}\leq i\leq\frac{N}{2}-1 \\ \left\{\begin{matrix} \mathbf{temp}_{1}=(\mathbf{K}[:,2i]+\mathbf{TD}_{\mathbf{1}}[:,2(i-1)])\mathrm{mod}256 \\ \mathbf{temp}_{2}=(\mathbf{K}[:,2i+1]+\mathbf{TD}_{\mathbf{1}}[:,2i-1])\mathrm{mod}256 \end{matrix}\right.\text{ if }1\leq i\leq\frac{N}{2}-1 \\ \left\{\begin{matrix} \mathbf{temp}_{1}=(\mathbf{K}[:,2i]+\mathbf{TD}_{\mathbf{2}}[:,2\times P_{2}])\mathrm{mod}256 \\ \mathbf{temp}_{2}=(\mathbf{K}[:,2i+1]+\mathbf{TD}_{\mathbf{2}}[:,2\times P_{2}+1])\mathrm{mod}256 \end{matrix}\right.\text{ if }i=0 \end{matrix}$ (31)

**Step 3**: Perform the column position restoration with the initial column diffusion position $P_{c}$, as follows:

$\mathbf{TD}_{2}[:,i]=\mathbf{TD}_{2}[:,N-1-(i-2\times P_{c})\mathrm{mod}N]$ 0≤ *i* ≤*N*-1 (32)

**Step 4**: Perform the first round of row restoration from the last two rows of **TD**_2_ (i.e., row *M*-1 and row *M*-2) to restore **TD**_3_, as follows:

$\begin{matrix} \left\{\begin{matrix} \mathbf{TD}_{3}[2i,:]=(\mathbf{temp}_{1}\oplus\mathbf{TD}_{2}[2i,:])\mathrm{mod}256 \\ \mathbf{TD}_{3}[2i+1,:]=(\mathbf{temp}_{2}\oplus\mathbf{TD}_{2}[2i+1,:])\mathrm{mod}256 \end{matrix}\right.\text{ 0}\leq i\leq\frac{M}{2}-1 \\ \left\{\begin{matrix} \mathbf{temp}_{1}=(\mathbf{K}[2i,:]+\mathbf{TD}_{2}[2(i-1),:])\mathrm{mod}256 \\ \mathbf{temp}_{2}=(\mathbf{K}[2i+1,:]+\mathbf{TD}_{2}[2i-1,:])\mathrm{mod}256 \end{matrix}\right.\text{ if }1\leq i\leq\frac{M}{2}-1 \\ \left\{\begin{matrix} \mathbf{temp}_{1}=(\mathbf{K}[2i,:]+\mathbf{TD}_{3}[M-1:,])\mathrm{mod}256 \\ \mathbf{temp}_{2}=(\mathbf{K}[2i+1,:]+\mathbf{TD}_{3}[M-2:,])\mathrm{mod}256 \end{matrix}\right.\text{ if }i=0 \end{matrix}$ (33)

**Step 5**: Perform the second round of row restoration from the last two columns of **TD**_3_ to obtain **TD**_4_, as follows:

$\begin{matrix} \left\{\begin{matrix} \mathbf{TD}_{4}[2\times(i+P_{r})\mathrm{mod}M,:]=(\mathbf{temp}_{1}\oplus\mathbf{TD}_{3}[2i,:])\mathrm{mod}256 \\ \mathbf{TD}_{4}[2\times(i+P_{r})\mathrm{mod}M+1,:]=(\mathbf{temp}_{2}\oplus\mathbf{TD}_{3}[2i+1,:])\mathrm{mod}256 \end{matrix}\right.\text{ 0}\leq i\leq\frac{M}{2}-1 \\ \left\{\begin{matrix} \mathbf{temp}_{1}=(\mathbf{K}[2i,:]+\mathbf{TD}_{3}[2(i-1),:])\mathrm{mod}256 \\ \mathbf{temp}_{2}=(\mathbf{K}[2i+1,:]+\mathbf{TD}_{3}[2i-1,:])\mathrm{mod}256 \end{matrix}\right.\text{ if }1\leq i\leq\frac{M}{2}-1 \\ \left\{\begin{matrix} \mathbf{temp}_{1}=(\mathbf{K}[2i,:]+IV_{3})\mathrm{mod}256 \\ \mathbf{temp}_{2}=(\mathbf{K}[2i+1,:]+IV_{4})\mathrm{mod}256 \end{matrix}\right.\text{ if }i=0 \end{matrix}$(34)

**Step 6**: Perform the row position restoration with the initial row diffusion position $P_{r}$, as follows:

$\mathbf{TD}_{4}[i,:]=\mathbf{TD}_{4}[M-1-(i-2\times P_{r})\mathrm{mod}M,:]$ 0≤ *i* ≤*M*-1 (35)

So far, the rewriting matrix $\mathbf{I}_{1}$ is restored, and $\mathbf{I}_{1}=\mathbf{TD}_{4}$.

Then, the plain image **I** is restored by performing a global rewriting restoration on the matrix $\mathbf{I}_{1}$, which is the reverse of the rewriting operation in formula (16).
